# Synthesis and multi-target antiproliferative evaluation of novel 1,2,4-triazole-3-thione analogues against breast cancer: *in silico* and *in vitro* mechanistic insights†

**DOI:** 10.1039/d5ra02512e

**Published:** 2025-07-14

**Authors:** Hussain Ali Almasmoum, Ghassan Almaimani, Riyad A. Almaimani, Abdullatif Taha Babakr, Maha Alsunbul, Hussah Abdullah Alshwyeh, Eman Serry Zayed, Ibrahim Abdel Aziz Ibrahim, Essa M. Saied

**Affiliations:** a Department of Clinical Laboratory Sciences, Faculty of Applied Medical Sciences, Umm Al-Qura University Makkah Kingdom of Saudi Arabia Haamasmoum@uqu.edu.sa; b Department of Surgery, Faculty of Medicine, Umm Al-Qura University Al Abdeyah, P. O. Box 7607 Makkah Saudi Arabia gamaimani@uqu.edu.sa; c Department of Biochemistry, Faculty of Medicine, Umm Al-Qura University Al Abdeyah Makkah 24381 Saudi Arabia Ramaimani@uqu.edu.sa atbabakr@uqu.edu.sa; d Department of Pharmaceutical Sciences., College of Pharmacy, Princess Nourah Bint Abdulrahman University P. O. Box 84428 Riyadh 11671 Saudi Arabia Maalsonbel@pnu.edu.sa; e Department of Biology, College of Science, Imam Abdulrahman Bin Faisal University 31441 Dammam Saudi Arabia haalshuyeh@iau.edu.sa; f Basic & Applied Scientific Research Centre, Imam Abdulrahman Bin Faisal University P. O. Box 1982 31441 Dammam Saudi Arabia; g Department of Clinical Biochemistry, Faculty of Medicine, University of Tabuk Tabuk Saudi Arabia ezayed@ut.edu.sa; h Department of Pharmacology and Toxicology, Faculty of Medicine, Umm Al-Qura University Makkah Saudi Arabia iamustafa@uqu.edu.sa; i Chemistry Department, Faculty of Science, Suez Canal University Ismailia 41522 Egypt essa.saied@science.suez.edu.eg; j Institute for Chemistry, Humboldt Universität zu Berlin 12489 Berlin Germany

## Abstract

Cancer remains a major global health challenge, with breast cancer representing the most commonly diagnosed malignancy in women. Heterocyclic scaffolds containing nitrogen and sulfur, such as 1,2,4-triazole-3-thione derivatives, have shown broad pharmacological utility, including notable anticancer potential. In the present study, a novel series of 4,5-disubstituted-1,2,4-triazol-3-thione derivatives was designed, synthesized, and fully characterized using NMR, IR, MS, and elemental analysis. The compounds were evaluated for their antiproliferative effects against MCF-7 (breast cancer) and HepG2 (liver cancer) cell lines. Most derivatives demonstrated notable cytotoxicity, with compound 6 exhibiting the most potent activity, achieving IC_50_ values of 4.23 μM and 16.46 μM against MCF-7 and HepG2 cells, respectively, comparable to the reference drug vinblastine. Mechanistic investigations revealed that compound 6 acts *via* a multi-targeted pathway, significantly inhibiting α-glucosidase (IC_50_ = 122.7 μM), tubulin-β polymerization (58.5% inhibition), and aromatase activity (31% reduction). Flow cytometry analysis confirmed that compound 6 induces pronounced S-phase cell cycle arrest and promotes both early and late apoptotic cell populations, along with a moderate increase in necrosis in MCF-7 cells. Additionally, compound 6 displayed dose-dependent antioxidant activity (DPPH IC_50_ = 25.4 μM), comparable to trolox. Complementary *in silico* studies provided molecular-level insights into the interactions of compound 6 with its biological targets. Molecular docking showed strong binding affinities through key hydrogen bonding and hydrophobic interactions within the active sites of tubulin, α-glucosidase, and aromatase. Molecular dynamics simulations over 100 ns confirmed the stability of these interactions, especially with α-glucosidase, supported by consistent RMSD, compactness, and favorable per-residue energy contributions. Overall, these findings identify compound 6 as a promising multi-target lead for further development as an anticancer agent, combining cytotoxic, enzyme-inhibitory, and antioxidant properties.

## Abstract 2

Deciphering the multi-target therapeutical potential of novel 1,2,4-triazole-3-thione analogues as potent antiproliferative agents against breast cancer.

## Graphical abstract

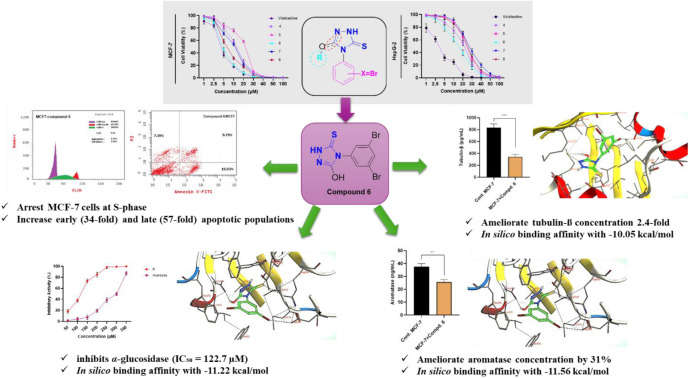

## 1. Introduction

Cancer remains one of the leading causes of mortality worldwide, with breast cancer being the most commonly diagnosed malignancy among women.^1,2^ Despite the current advancements in cancer treatment including targeted therapies, chemotherapy, and immunotherapy, there are still major challenges that persist such as multidrug resistance, off-target toxicity, and severe side effects. Therefore, there is an urgent demand for the discovery of novel anticancer therapeutic strategies.^3^ Recently, attention has been directed toward the ‘multi-target therapeutic’ strategies that concurrently interrupt various biological pathways or “hallmarks” of cancer, including cell proliferation, hormonal regulation, metabolic pathways, and oxidative stress. By interfering with cancer progression at several nodes, a multi-target strategy may hinder tumor growth, ultimately leading to more durable responses. Accordingly, the discovery of novel bioactive compounds capable of targeting multiple cancer-relevant pathways has become an attractive and promising strategy for cancer treatment.^4^ In the context of cancer hallmarks, different targets have been incorporated with distinct aspects in tumor growth and survival, including tubulin-β, aromatase, and α-glucosidase. Tubulin-β, a structural subunit of microtubules, is essential for mitotic spindle formation and cell division. Inhibiting tubulin-β polymerization causes mitotic arrest and apoptosis in cancer cells, making it a prime cytoskeletal target in cancer therapy.^5,6^ Aromatase, on the other hand, is a key enzyme in estrogen biosynthesis (catalyzing the final step of estrogen production) and is crucial for the growth of hormone-dependent tumors. Aromatase inhibitors (*e.g.* letrozole, anastrozole) are well-established treatments for estrogen receptor-positive breast cancer, effectively suppressing estrogen levels to hinder tumor growth.^7^ α-Glucosidase is an enzyme involved in glycoprotein processing, with growing evidence for its relevance in cancers. Recent studies showed that suppressing lysosomal α-glucosidase impairs cancer cell metabolism, enhances chemosensitivity, and induces apoptosis.^8^ Thus, a compound that inhibits α-glucosidase may exhibit a potential anticancer activity by targeting glucose deprivation and glycolysis.^9,10^ Another facet of cancer progression is the dysregulation of reactive oxygen species and oxidative stress. Cancer cells typically sustain higher basal reactive oxygen species (ROS) levels than normal cells, ultimately promoting DNA mutations that drive tumorigenesis. Compounds with antioxidant properties might therefore potentially improve the redox balance and mitigate oxidative stress in cancer cells inhibiting the malignant progression.^11,12^

**Fig. 1:**
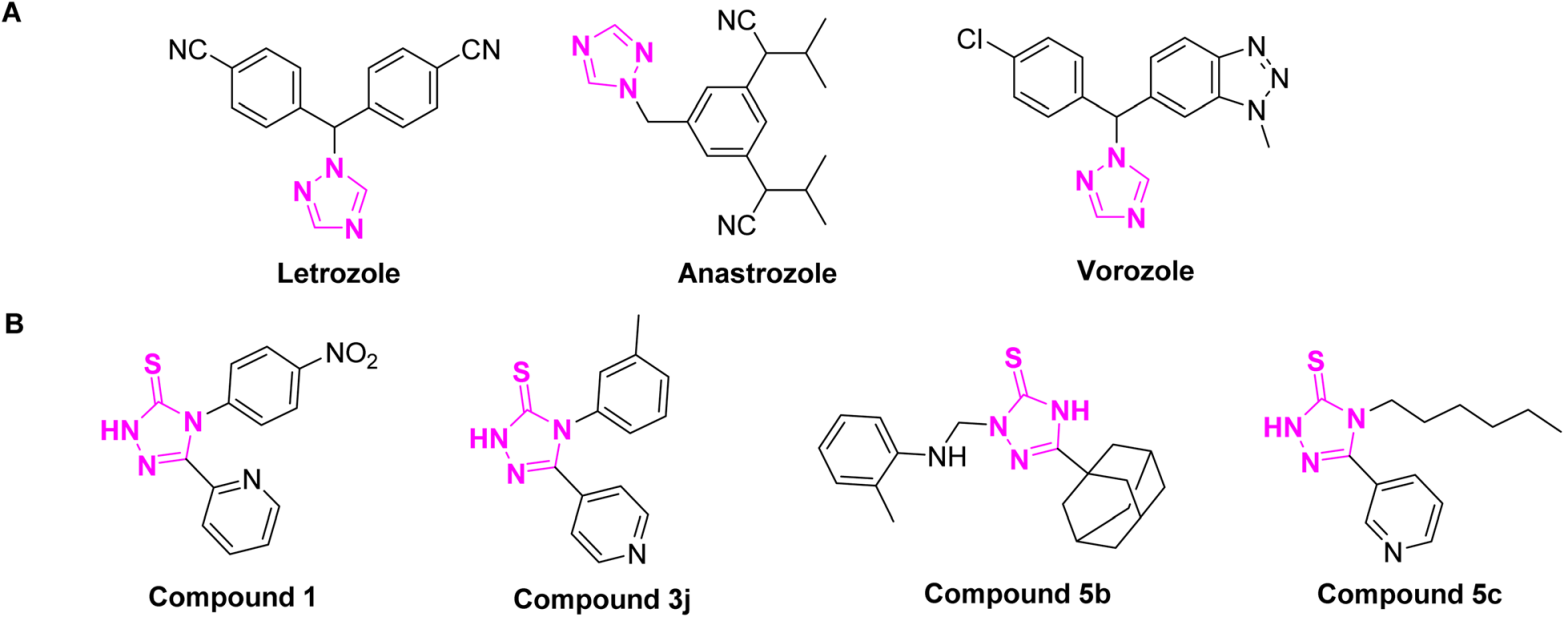
Representative structures of bioactive 1,2,4-triazole derivatives. (A) Structures of clinically approved anticancer drugs with 1,2,4-triazole moiety. (B) structures of previously reported 1,2,4-triazole-3-thione derivatives with potential antiproliferative activity and act as multi-functional agents.

Triazoles are five-membered heterocycles that have a privilege in medicinal chemistry owing to their synthetic accessibility, chemical versatility, and biological activity.^13^ Among triazoles, the 1,2,4-triazole-3-thione scaffold has emerged as a crucial scaffold of several therapeutically bioactive compounds with versatile pharmacological activities, including anticancer, antimicrobial, anti-inflammatory, anticonvulsant, antiepileptic, anti-HIV, and antitubercular.^14–19^ This scaffold has been widely explored in the design of anticancer agents due to its ability to interact with diverse biomolecular targets, including enzymes, receptors, and DNA. The thione (–C=S) and triazole nitrogen atoms offer multiple binding sites (*e.g.* hydrogen bonding and metal coordination) that enhance affinity toward various proteins, enabling this moiety to act as a pharmacophore in enzyme inhibition, apoptosis induction, and cell cycle modulation.^16,17,19^ A number of FDA-approved anticancer drugs, such as letrozole, anastrozole, and vorozole, already incorporate a 1,2,4-triazole moiety, highlighting its relevance in drug development (Fig. 1A). Several studies have demonstrated the antiproliferative effects of 1,2,4-triazole-3-thione derivatives against various cancer cell lines including breast, liver, lung, and leukemia models.^16,17^ Beyond their antiproliferative effects, 1,2,4-triazole-3-thione derivatives have been identified to act *via* specific mechanisms implicated in cancer progression including enzyme inhibition, apoptosis induction, or cell cycle arrest. Pitucha *et al.* developed a series of triazolin-5-thione analogues, including compound 1 (Fig. 1B), which demonstrated notable antiproliferative activity by specifically inhibiting casein kinase 1γ. This kinase is involved in regulating Wnt/β-catenin signaling, a pathway critical to tumor growth and survival. Inhibition of CK1γ by these analogues provides a novel approach to cancer treatment by disrupting downstream transcriptional activation of cell survival genes.^20^ In a separate study, Bhat *et al.* investigated 4-aryl-5-(pyridin-4-yl)-3*H*-1,2,4-triazole-3-thiones and reported that compound 3j induced apoptosis in cancer cells *via* caspase-3 activation. This was attributed to the compound's ability to initiate mitochondrial membrane depolarization, leading to cytochrome c release and triggering the intrinsic apoptotic cascade.^21^ Similarly, Milošev *et al.* explored a class of 5-adamantyl-substituted 1,2,4-triazole-3-thione derivatives and highlighted compound 5b, which exhibited both potent and selective antiproliferative activity across various cancer cell lines. Mechanistically, this compound induced cell cycle arrest in the sub-G_1_/G_1_ phase and modulated apoptotic pathways through caspase activation, indicating interference with DNA replication or damage response checkpoints.^22^ Additionally, Nadeem *et al.* reported compound 5c, a triazole-based molecule with significant free radical scavenging activity. Its antioxidant capability was associated with reduced intracellular ROS levels, offering a dual benefit by protecting normal cells and attenuating oxidative stress-induced tumor progression.^23^ These findings collectively demonstrate the multifaceted biological activities of 1,2,4-triazole-3-thione derivatives, particularly their antiproliferative effects and enzyme inhibitory properties, suggesting them as promising candidates for the development of multi-functional therapeutic agents for addressing the ongoing challenges in cancer treatment.

**Fig. 2:**
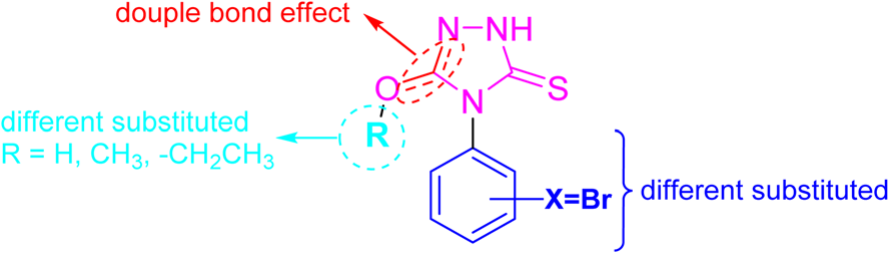
Illustration of the design of envisioned structural features of 4,5-disubstituted-1,2,4-triazol-3-thione derivatives.

Based on these facts and our ongoing efforts toward the discovery of new antitumor agents,^24–29^ the presented study reported the synthesis and characterization of a set of novel 4,5-disubstituted-1,2,4-triazol-3-thione derivatives. The structure of the envisioned compounds was designed to examine various structural features around the 1,2,4-triazol-3-thione scaffold through strategic substitutions at positions 4 and 5 of the triazole ring, introducing functional groups that could enhance binding affinity and cellular permeability (Fig. 2). We further aimed to explore the cytotoxic activity of envisioned compounds against HepG2 and MCF-7 cells, but also to unveil their multi-target mode of action. In this regard, we envisioned investigating their activity toward cell cycle arrest, induction of programmed cell death, and apoptotic-related targets, but also to address key molecular pathways implicated in cancer progression. Toward this, we rationally selected three enzymes for evaluation: tubulin-β, essential for mitotic spindle formation and cell division; aromatase, critical in estrogen biosynthesis and thus in hormone-dependent tumor growth; and α-glucosidase, which has recently been linked to cancer cell metabolism and glycoprotein processing. As such, this study not only expands the chemical diversity of 1,2,4-triazole-3-thione derivatives but also validates their role as promising multitarget agents against cancer.

## 2. Results and discussion

### 2.1. Chemistry

**Scheme 1:**
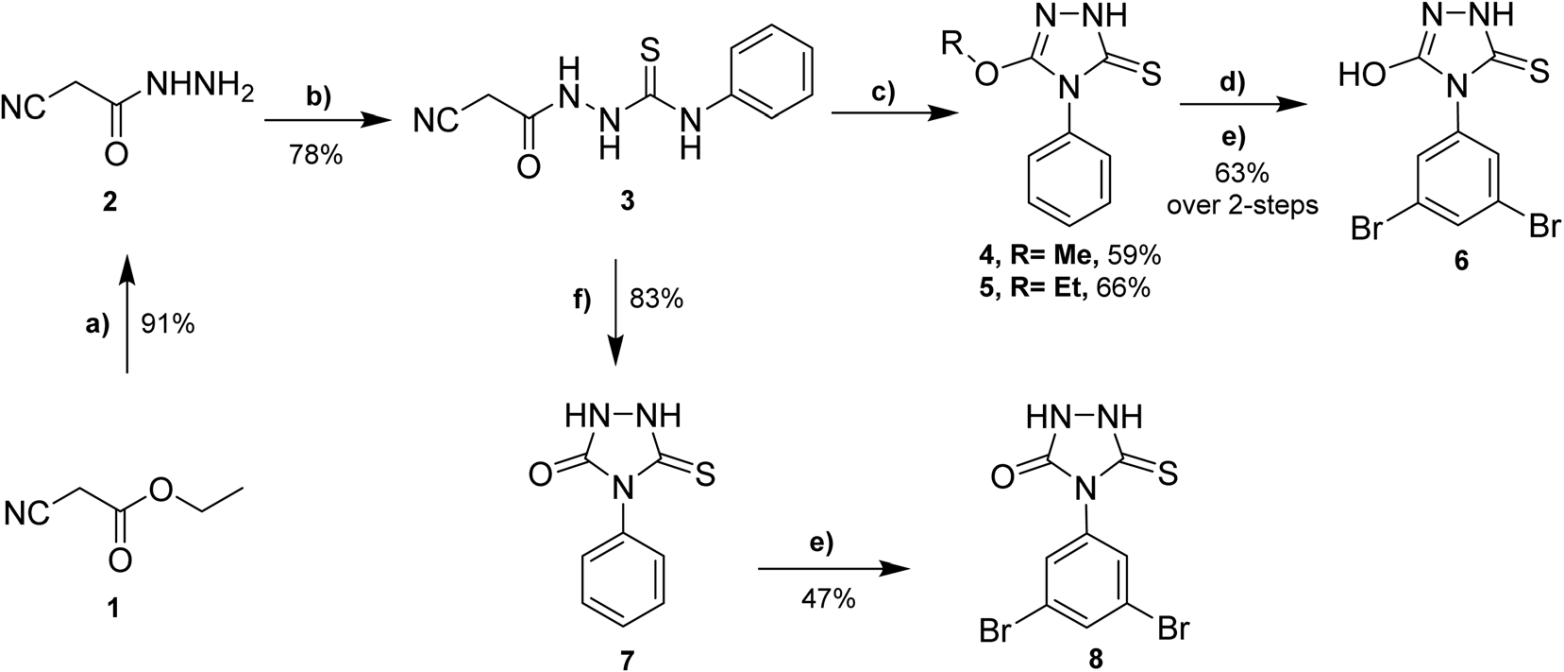
Syntheses of 4,5-disubstituted-1,2,4-triazol-3-thione derivatives 4–8. Reagent and conditions: (a) hydrazine, EtOH, 16 h, reflux; (b) phenyl isothiocyanate, benzene, 8 h, reflux; (c) NaOH in MeOH or EtOH, 12–16 h, reflux; (d) 48% HBr, 10 mol% TBAB, acetic acid, 12 h, 110 °C; (e) Br_2_, alcetic acid, 7–10 h, reflux; (f) 10% aqueous NaOH, 12 h, reflux.

The synthesis of envisioned 4,5-disubstituted-1,2,4-triazol-3-thione derivatives 4–8 was achieved following the synthetic route presented in Scheme 1, starting from commercially available ethyl cyanoacetate (1). The synthesis started with the condensation of ethyl cyanoacetate 1 with hydrazine hydrate in ethanol to afford cyanoacetic acid hydrazide 2 in quantitative yield.^30^ The subsequent reaction of compound 2 with phenyl isothiocyanate under reflux provided the key intermediate 1-cyanoacetyl-4-phenylthiosemicarbazide (3) in considerable yield (78%).^31^ The cyclization of intermediate 3 to 1,2,4-triazol ring was successfully achieved by reaction with alcoholic (methanolic or ethanolic) sodium hydroxide solution to afford 5-alkoxy-4-phenyl-1,2,4-triazol-3(2*H*)-thione 4 and 5 in satisfactory yields, respectively. To further extend our explorations, we envisioned investigating the effect of alkoxy substitution on triazole ring, but also the effect of halide substitution on the aromatic ring. Toward this, compound 4 reacted with 48% hydrogen bromide in the presence of catalytic tetrabutylammonium bromide (TBAB), as a phase transfer catalyst, to afford the corresponding demethylated 5-hydroxy analogue.^32,33^ To explore the structural feature at the aromatic ring, the obtained 5-hydroxy analogue was reacted with bromine in an acidic solution to provide the 3,5-dibromophenyl-5-hydroxy-1,2,4-triazole-3-thione 6 in a good yield (63%, over 2 steps). On the other hand, to unveil the structural features of the substitution at position 5 in the 1,2,4-triazole ring, we envisioned extending our synthesis to obtain the 5-oxo-substituted 1,2,4-triazol-3-thione scaffold. Thus, the reaction of compound 3 with 10% aqueous sodium hydroxide solution successfully led to the formation of the 5-oxo-1,2,4-triazol-3-thione 7 in 83% yield. Finally, the reaction of compound 7 with an acidic solution of bromine resulted in the formation of the 5-oxo-4-(3,5-dibromophenyl)-1,2,4-triazol-3-thione 8 (47% yield).

**Fig. 3:**
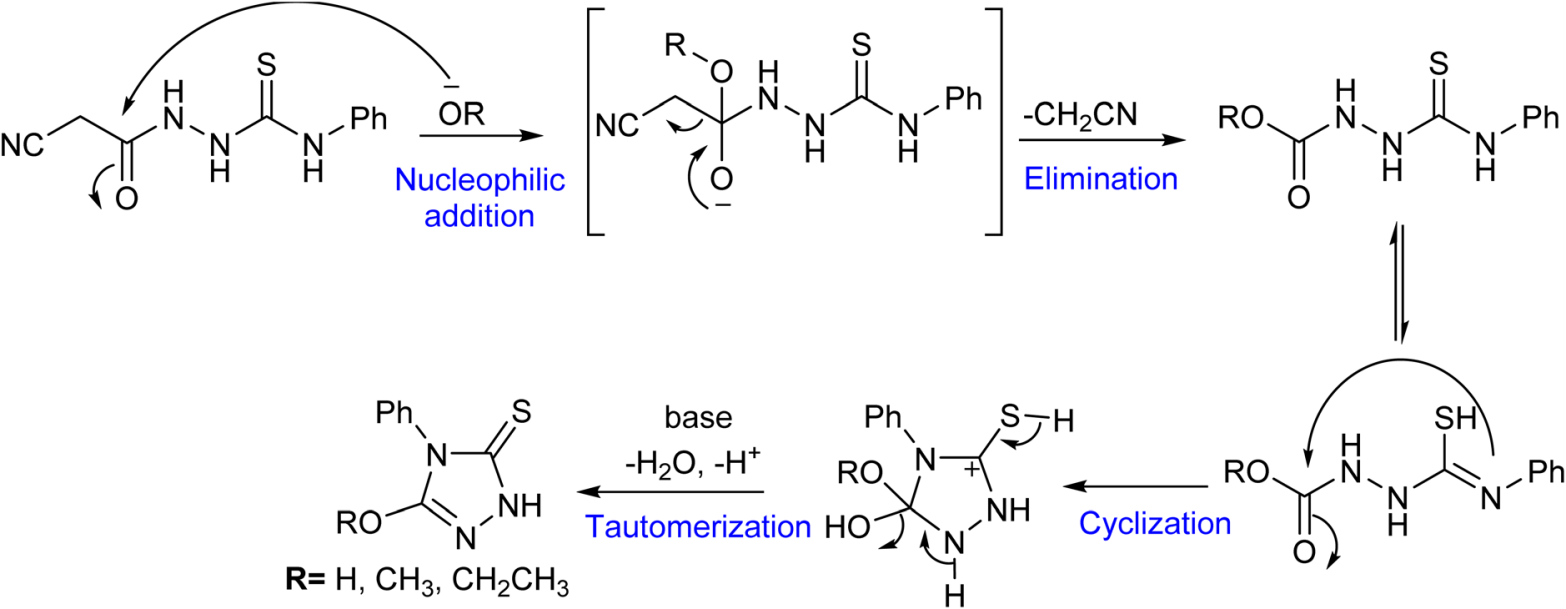
The proposed mechanism for the formation of the 1,2,4-triazole-3-thione compounds.

The formation of 1,2,4-triazole-3-thione derivatives could be attributed to proceeding through a sequence of nucleophilic addition, elimination, cyclization, and tautomerization.^34–37^ As shown in Fig. 3, the reaction initiates with the nucleophilic attack of the alkoxy (^−^OR) or hydroxy (^−^OH) moiety on the carbonyl carbon, forming a transient hydrazone intermediate. This intermediate undergoes activation, facilitating the elimination of a cyanomethylene (–CH_2_CN), which is stabilized by resonance delocalization. Following elimination, intramolecular cyclization occurs, where the nitrogen of the thiourea group attacks an electrophilic center of the carbonyl ester, leading to the closure of the five-membered 1,2,4-triazole ring. The final transformation involves tautomerization, in which the sulfur atom rearranges to form a stable thione (–C=S) functional group, completing the synthesis.^35,37,38^

Several analytical analyses have been applied to elucidate the structure of compounds including mass spectrometry, FTIR, NMR, and elemental analysis. The study of ^1^H-NMR spectra of compound 4 revealed a singlet signal at *δ* 4.00 ppm which was assigned for three protons of the methoxy (OCH_3_) group, while in compound 5, ^1^H NMR spectrum showed two signals at *δ* 4.52–4.55 ppm (quartet) and *δ* 1.30–1.34 ppm (triplet) referring to the ethoxy (OCH_2_CH_3_) group. The aromatic ring (CH=CH) protons of compounds 4 and 5 were detected in the estimated region 7.14–7.65 as three signals for five protons. The NH signal in compounds 4 and 5 demonstrated singlet signals at *δ* 11.13 and 11.06 ppm, respectively. The C-3 and C-5 of the triazole ring of compounds 4 and 5 were demonstrated by the carbon signals at *δ* 188.9 and 139.0 ppm in the ^13^C NMR spectra for compound 4 and at *δ* 187.8 and 138.9 ppm for compound 5. Also, compound 4 showed a carbon signal at *δ* 58.2 ppm which corresponds to the methoxy (OCH_3_) group, while compound 5 exhibited the ethoxy group carbon signals (OCH_2_CH_3_) at *δ* 67.6 and 14.4 ppm. On the other hand, the spectrum of compound 6 displayed the absence of the proton and carbon signals of the methoxy (OCH_3_) group, which are presented in compound 4. Further, compound 6 demonstrated 2-singlet signals for the aromatic-H at *δ* 8.05 (for two protons) and *δ* 7.88 ppm (for one proton), demonstrating the di-bromination of compound 4. The spectrum of compound 6 exhibited two carbon signals at *δ* 189.9 and 156.5 which were assigned to the C-3 and C-5 of the triazole ring. Additionally, the aromatic ring signals were observed at *δ* 134.5 (N–C=C), 130.9 (2× C–Br), and 112.4 ppm.

The assessment of the mass fragmentation pattern of compounds 5–7 was also performed. As illustrated in Scheme S1,† compound 5 displayed a molecular ion peak at *m*/*z* 221 which is associated with the molecular formula C_10_H_11_N_3_OS. The *m*/*z* 221 ion further fragmented by losing the ethylene molecule to afford a signal at *m*/*z* 193, corresponding to the molecular ion peak of compound 7. The loss of the NCS group gave an ion peak at *m*/*z* 135. The common peak observed at *m*/*z* 93 could be attributed to the loss isocyanate (N=C=O) group from the ion of *m*/*z* 135. The stable *m*/*z* 93 ion peak was further fragmented by the loss of the H-atom and acetylene molecule to provide signals at *m*/*z* 92 and 66, respectively (Scheme S1†). Further, the *m*/*z* 221 molecular ion underwent loss of nitrogen atom to give a peak at *m*/*z* 207, which further fragmented to produce an ion at *m*/*z* 135. The loss of isothiocyanate (N=C=S) group from the ion with *m*/*z* 135 resulted in an ion at *m*/*z* 77. On the other hand, compound 6 exhibited a molecular ion peak of *m*/*z* 352/349 and was unstable. The molecular ion peak underwent fragmentation to provide a stable ion peak at *m*/*z* 291/289 by losing the NH_2_–C^+^=S group. The loss of hydrogen bromide (HBr) and bromine atom from *m*/*z* 291/289 provided ion peaks at *m*/*z* 211/209 and 130, respectively (Scheme S1†).

### 2.2. Assessment of antiproliferative activity

**Fig. 4:**
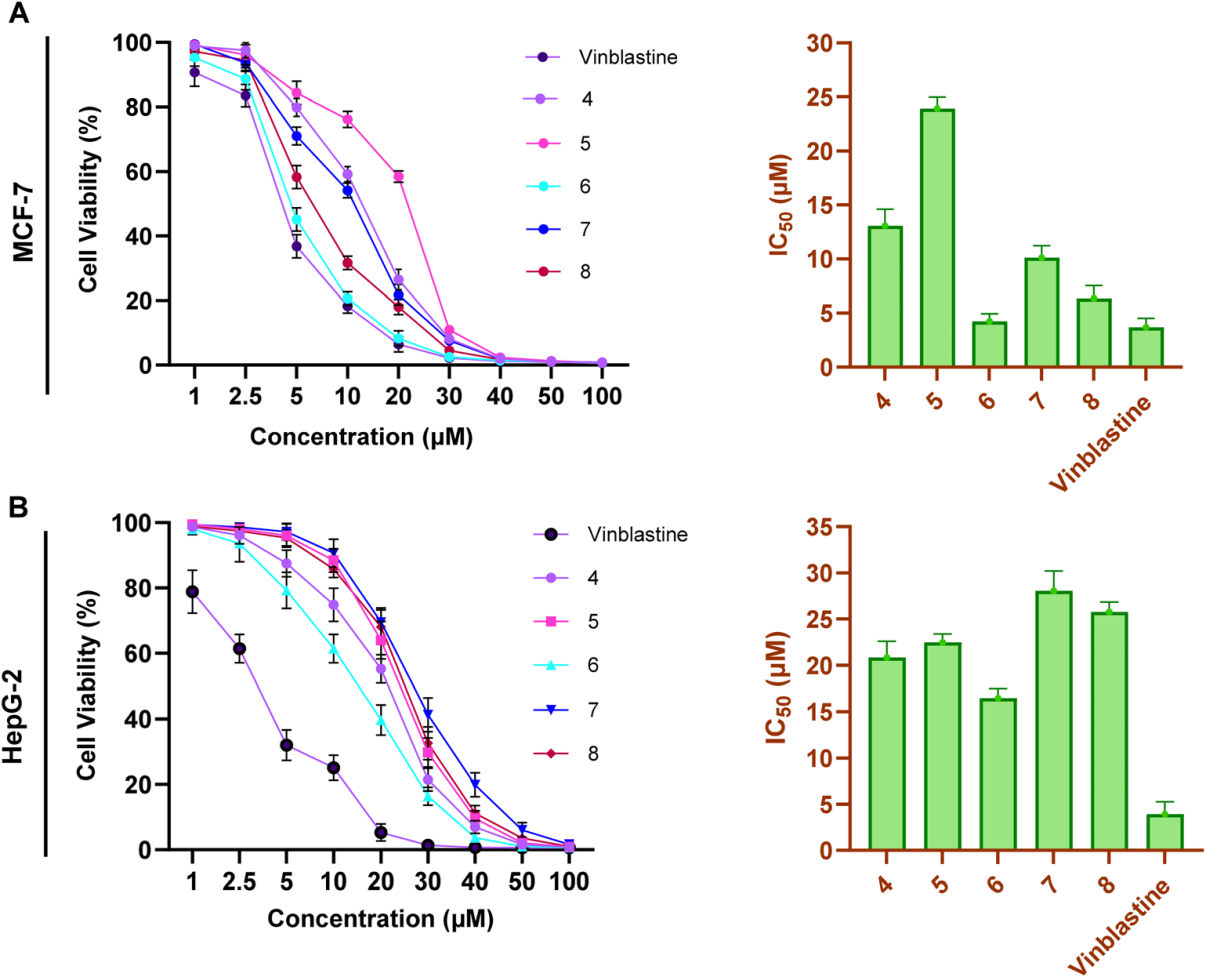
The dose-dependent activity and IC_50_ values of compounds 4–8 and vinblastine toward the cell viability of MCF-7 (A) and HepG-2 (B) cells. The presented data is displayed as mean ± SD, *n* = 3.

We first envisioned exploring the cytotoxic activity of the synthesized compounds (4, 5, 6, 7, and 8) toward the MCF-7 (human breast cancer) and HepG2 (liver carcinoma) cell lines utilizing the well-established MTT assay to broadly assess the antiproliferative potential of the synthesized compounds across distinct cancer types. In this regard, we evaluated the antiproliferative activity of examined compounds at different concentrations (1, 2.5, 5, 10, 20, 30, 40, 50, and 100 μM) and applied the anticancer drug Vinblastine as a reference drug for our assessments.^39,40^ As shown in Fig. 4, the examined compounds exhibited substantial and dose-dependent cytotoxic activity toward the MCF-7 cells with IC_50_ values ranging from 4.32 to 23.93 μM. The assessment of the structural-activity relationship (SAR) revealed that the cytotoxic activity of this class of compounds was significantly influenced by the substitution at positions 4 (aryl group), and 5 (R group) in the 1,2,4-triazol-3-thione moiety. The alkylation of the 1,2,4-triazol-3-thione moiety at position 5 with either methyl or ethyl-group resulted in compounds that display moderate cytotoxicity (compound 4, IC_50_ = 13.07 μM; and compound 5, IC_50_ = 23.93 μM). These results indicate that the electron-donating property of the alkyl groups may either impair the electrophilic nature of the triazole ring or cause a steric hindrance, diminishing interactions with biological target (s). This hypothesis was supported by the observed enhanced cytotoxicity of compound 4 (methyl group) as compared to that of compound 5 (ethyl group). The replacement of alkyl group with carbonyl group at position 4 provided a compound (compound 7) with substantially boosted cytotoxic activity (IC_50_ = 10.11 μM), further supporting that the presence of alkyl group at position 4 diminishing the antiproliferative activity of this class of compound. Bromination of the aryl moiety at position 5 in compound 7 significantly improved the cytotoxicity of the compound (compound 8, IC_50_ = 6.36 μM). These results indicate that the presence of bromine atoms at the *meta*-positions of the aryl group augments the interaction with biological target(s), possibly through π–π stacking interactions, improved lipophilicity, or electronic modulation, thereby leading to better cytotoxicity. Interestingly, the replacement of the carbonyl group (H-acceptor) in compound 8 with a hydroxy group (H-donor) resulted in a significant improvement in the antiproliferative activity of compound (compound 6, IC_50_ = 4.23 μM), indicating that the hydroxyl group plays a potential role to enhance the interactions with the cellular target(s), potentially *via* hydrogen bonding. Notable, among the examined compounds, compound 6 displayed a comparable the most potent cytotoxic effect toward MCF-7 cells as comparable to that of the vinblastine drug (IC_50_ = 3.68 μM).

On the other hand, the assessment of the cytotoxic activity of examined compounds toward HepG-2 cells revealed that this class of compounds possess less antiproliferative activity, as compared to MCF-7 cells, with IC_50_ values in the range of 16.46 to 28.04 μM. The activity of all examined compounds was less than that of vinblastine (IC_50_ = 3.92 μM), suggesting that these compounds may be less efficient in liver carcinoma cells due to differences in cellular metabolism (Fig. 4). Evaluation of the SAR analysis revealed that both substituents at position 4 (alkyl group) and position 5 (aryl group) considerably impact the cytotoxic activity of this class. In contrast to that in MCF-7 cells, the SAR study indicated that the compounds with alkyl group at position 4 exhibit considerable cytotoxicity (compounds 4 and 5, IC_50_ = 20.84 μM, 22.47 μM, respectively). The replacement of alkyl group with carbonyl group led to a considerable amelioration in the antiproliferative activity of the resulting compound, as indicated with compound 7 (IC_50_ = 28.04 μM). Nevertheless, the bromination of compound 7 at the *meta*-position of the aryl group resulted in the augmentation of cytotoxic activity as noticed by compound 8 (IC_50_ = 25.77 μM). Similarly, the replacement of carbonyl group in compound 8 with the H-donating group (hydroxyl group) led to a significant enhancement in the antiproliferative activity of the compound (compound 6, IC_50_ = 16.46 μM). Together, our analysis revealed that this class of compounds holds considerable antiproliferative activity, particularly against MCF-7 cells. The SAR analysis implies that H-donating substituents at position 4, particularly hydroxyl group, and the presence of an electron-withdrawing 3,5-dibromophenyl ring at position 5 contributes to increased cytotoxicity. Encouraged by these findings, we extended our investigations to explore the selectivity of this class of compounds toward cancer cells. Toward this, we assessed the antiproliferative activity of compounds 4–8 toward the normal breast MCF-10A cells. Our results revealed that all compounds possessed non-considerable cytotoxic effects on MCF-10A cells with IC_50_ > 20 μM (Fig. S8†). The exploration of selectivity index values revealed that, except compound 5, all compounds displayed considerable selectivity index value (SI > 2), as compared to that of MCF-7 cells. Furthermore, our findings indicate that this class of compounds exhibits insignificant toxicity toward normal cells as compared to the known anticancer vinblastine. Among compounds, compound 6 emerged as the most potent derivative, showing comparable cytotoxicity to vinblastine against MCF-7 cells, moderate efficacy in HepG2 cells, and considerable selectivity index as compared to that of MCF-10A cells. We acknowledge that further validation in additional breast cancer subtypes (*e.g.*, triple-negative or HER2-positive lines) is warranted and future efforts should be directed to extend the scope and translational relevance of these findings.

### 2.3. Evaluation of α-glucosidase activity

**Fig. 5:**
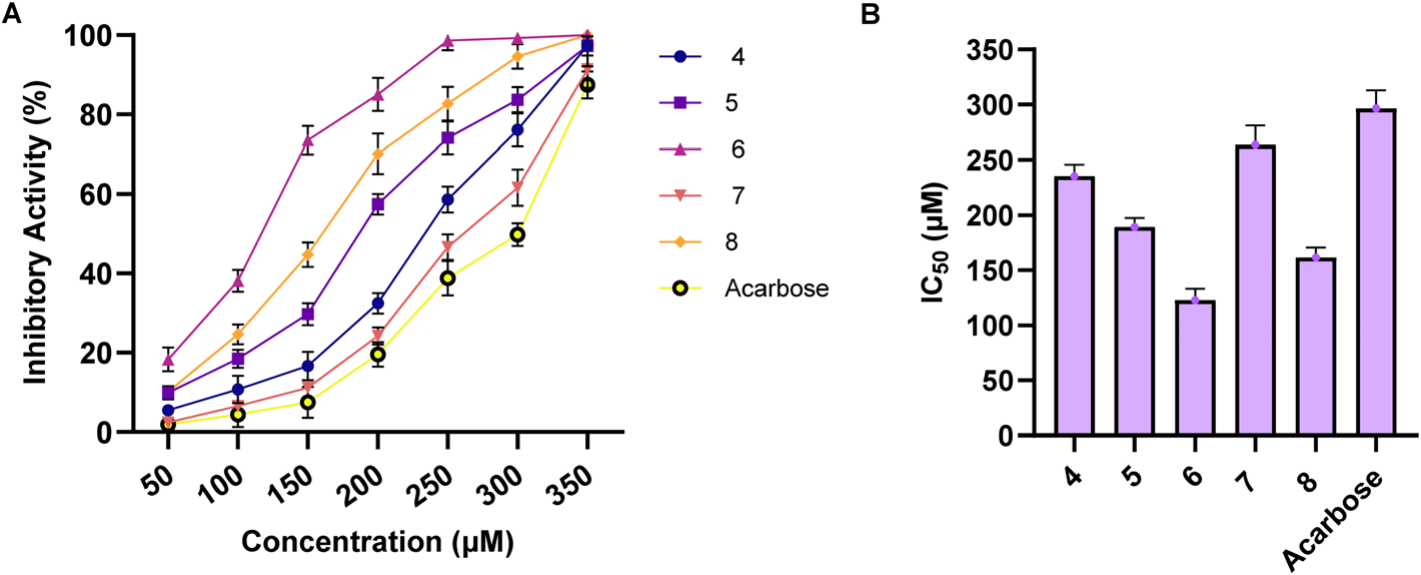
The inhibitory activity of newly synthesized 4,5-disubstituted 1,2,4-triazol-3-thiones analogues 4–8 and acarbose toward α-glucosidase enzyme. (A) The dose-dependent inhibitory activity of compounds 4–8 in comparison to the acarbose toward α-glucosidase enzyme. (B) The detected IC_50_ values of compounds 4–8 against α-glucosidase activity compared to the drug acarbose. The presented data is displayed in triplicate as mean ± SD.

Neutral α-glucosidases play a pivotal role in glycoprotein processing and maturation, which are essential metabolic processes in cancer progression. Targeting these glucosidases has emerged as a potential strategy to disrupt tumor-associated angiogenesis, cell migration, and overall tumor growth. Therefore, α-glucosidase inhibition could serve as an adjunct mechanism complementing direct cytotoxicity toward cancer cells.^41,42^ Based on the promising antiproliferative activity of the synthesized compounds, we extended our investigations to explore whether the cytotoxicity of the compounds is associated with their activity toward α-glucosidase. Our results indicated the examined compounds exhibited a potential and dose-dependent inhibitory activity toward α-glucosidase, with IC_50_ values in the range of 122.7 to 263.4 μM surpassing that of the α-glucosidase acarbose inhibitor (IC_50_ = 296.6 μM). As shown in Fig. 5, compound 6 demonstrated the most potent inhibition with an IC_50_ of 122.7 μM, followed by compound 8 (IC_50_ = 161.3 μM) and compound 5 (IC_50_ = 189.1 μM). The remaining compounds, 4 and 7, exhibited weaker inhibitory effects with IC_50_ values of 235.2 μM and 263.4 μM, respectively. Examination of the SAR analysis revealed that compounds 4 and 5 with an electron-donating alkoxy group (OCH_3_ and OCH_2_CH_3_, respectively) at position 4 exhibit considerable inhibitory activity. Replacement of alkoxy group with carbonyl group led to impairment of inhibitory activity, as indicated with compound 7, indicating an electron-withdrawing group at this position may reduce the affinity toward the enzyme. Nevertheless, bromination of the aryl-moiety at *meta* positions significantly improved the inhibitory potency of the compound, as indicated for compound 8. These results indicate that electron-withdrawing halogens enhance enzyme binding by increasing lipophilicity, enabling better interactions within the hydrophobic pocket of the enzyme. In agreement with these results, previous studies showed that 1,2,4-diaryl-substituted triazole-3-thiones tend to be potent, whereas those lacking an aromatic substitution are weaker.^43^ Interestingly, the replacement of carbonyl group at position 4 in compound 8 with hydroxyl group led to the most potent analogue, compound 6 (IC_50_ = 122.7 μM), suggesting that hydroxylation enhances enzyme binding, possibly through hydrogen bonding interactions with active site residues. These results highlight the advancement of our presented 1,2,4-trialzole analogues as compared to reported analogues. Previous studies on related 1,2,4-triazole compounds reported α-glucosidase inhibitors with IC_50_ values ranging from around 202 μM to over 800 μM.^44^ Overall, our investigations indicated that the newly synthesized class of compounds exhibits extensive inhibitory activity toward α-glucosidase. Among this class, compound 6 displayed the most potent inhibitory activity, together with considerable binding affinity toward the pocket. These findings indicate that the potent antiproliferative activity of compound 6 is associated with its ability to dual target cell proliferation and interfere with glycoprotein processing through α-glucosidase inhibition, potentially impeding processes like angiogenesis and metastasis alongside directly inducing cancer cell death.

### 2.4. Assessment of cell cycle arrest and programmed cell death

**Fig. 6:**
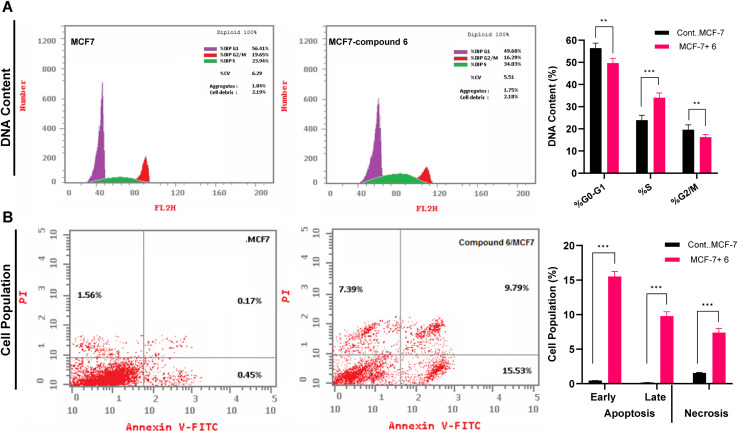
Impact of compound 6 on cell cycle dynamics and induction of programmed cell death in MCF7 cells. (A) The effect of compound 6 at 4.23 μM on the distribution of DNA content in the G_0_/G_1_, S, and G_2_/M phases of MCF-7 cells after 24 h as detected by flow cytometry. (B) The effect of compound 6 at 4.23 μM on the induction of apoptosis and necrosis in MCF-7 cells after 24 h as detected by Annexin V-FITC assay. The presented data is displayed in triplicate as mean ± SD. Differences between experimental groups were assessed by Student's *t*-tests and considered significant with *p* ≤ 0.05 (*p ≤ 0.05, **p ≤ 0.01, and ***p ≤ 0.001).

Given that compound 6 exhibited a dual antiproliferative mechanism with potent cytotoxic activity and α-glucosidase inhibition, we hypothesized that its ability to interfere with glycoprotein processing could contribute to impaired cell proliferation, apoptosis induction, and potential inhibition of tumor progression. Toward this, we aimed to further explore the effect of compound 6 on cell cycle arrest and induction of programmed cell death in MCF-7 cells. The effect of compound 6 on cell cycle progression was analyzed by flow cytometry analysis of DNA content in MCF-7 cells after 24-hour treatment at its IC_50_ concentration (4.23 μM). As shown in Fig. 6, our results revealed that treatment with compound 6 led to a significant enhancement in the DNA content in the S-phase, with an increase from 23.94% (untreated) to 34.03% (treated), accompanied by a significant reduction in both the G_0_/G_1_ (from 56.41% to 49.68%) and G_2_/M (from 19.65% to 16.29%) populations. These findings indicate that compound 6 induces S-phase arrest, suggesting that it interferes with DNA synthesis and replication rather than mitotic progression, potentially through targeting DNA polymerases and/or replication-associated targets. The amelioration of population in both G_0_/G_1_ and G_2_/M phases further suggests that cells are unable to complete replication and progress efficiently through the cell cycle. In agreement with our findings, various studies showed that 1,2,4-triazole-3-thione analogues demonstrate the potential antiproliferative activity by targeting the cell cycle at various checkpoints in different cancer cells.^45–48^ Given the observed potent inhibitory activity toward α-glucosidase, our findings suggest that compound 6 may interfere with key pathways influencing DNA replication by targeting glycoprotein metabolism, eventually leading to programmed cell death.

To assess whether S-phase arrest leads to apoptosis, we further performed Annexin V-FITC/PI staining to assess apoptotic and necrotic cell populations. As shown in Fig. 6, after treatment of MCF-7 with compound 6 at 4.23 μM for 24 h, a significant increase in early (34-fold) and late (57-fold) apoptotic cell populations were observed as compared to untreated cells. These findings indicate that compound 6 triggers intrinsic apoptosis at different stages in MCF-7 cells, likely through mitochondrial dysfunction metabolic disruption, and activation of caspase signaling pathways. Further, a considerable increase in necrotic cell death (5-fold) was revealed, suggesting that while apoptosis is the primary response, a subset of cells undergoes necrosis, possibly due to excessive DNA damage or metabolic disruption associated with α-glucosidase inhibition. The relatively low necrosis rate compared to apoptosis indicates that compound 6 predominantly induces apoptosis rather than nonspecific necrotic cell death. Further, the progression from early to late apoptosis implies that compound 6 triggers mitochondrial outer membrane permeabilization, cytochrome c release, and caspase activation, resulting in apoptotic cell death.^49,50^ Several 1,2,4-triazole-3-thione derivatives have been reported to induce apoptosis in cancer cells, often through mitochondrial dysfunction and oxidative stress.^45–48^ Compared to previously reported analogs, compound 6 uniquely combines S-phase arrest with α-glucosidase inhibition, making it a distinct candidate in anticancer therapy. Together, our findings indicate that compound 6 exerts a dual-action antiproliferative mechanism by impairing both DNA replication and glycoprotein metabolism leading to inhibit cancer cell proliferation and induce intrinsic apoptosis.

### 2.5. Assessment of DPPH free radical scavenging activity

**Fig. 7:**
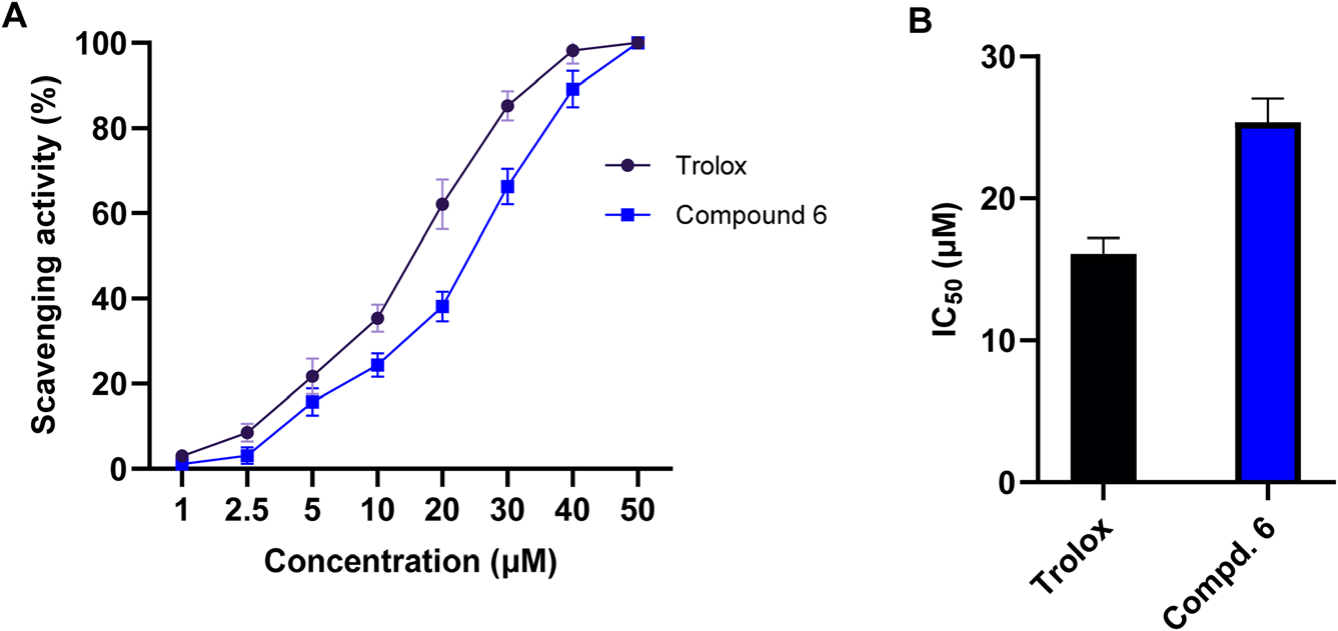
The free radical scavenging activity of compound 6 toward DPPH radicals. (A) The dose-dependent scavenging activity of compound 6 and Troxol standard toward DPPH radicals. The presented data is displayed in triplicate as mean ± SD. (B) Representation of the IC_50_ values of compound 6 and Troxol toward DPPH radicals.

Free radicals, particularly ROS, play a critical role in cancer development and progression by inducing oxidative stress, DNA damage, and inflammation. Antioxidants counteract these harmful effects by neutralizing free radicals, thereby reducing oxidative stress and protecting cellular integrity.^51,52^ To explore the potential antioxidant activity of compound 6, we extended our investigations to explore its free radical scavenging capacity using DPPH (2,2-diphenyl-1-picrylhydrazyl) assay. In our study, we examined the scavenging activity of compound 6 at different concentrations (1, 2.5, 5, 10, 20, 30, 40, and 50 μM) and compared the results to the reference antioxidant drug, Trolox (a water-soluble vitamin E analog). As shown in Fig. 7, our results showed that compound 6 exhibits a substantial, dose-dependent DPPH radical scavenging activity, with an IC_50_ of 25.4 μM. Interestingly, this activity was comparable to that of Trolox (IC_50_ = 16.1 μM), demonstrating that compound 6 possesses strong antioxidant properties. The observed antioxidant activity of compound 6 can be attributed to its structural features including the hydroxyl (–OH), amino (–NH), and thione (–C=S) groups, which enable radical-scavenging efficiency, stabilizing free radicals and reducing oxidative stress. Overall, these findings highlight compound 6 as a potential dual-functional therapeutic agent, combining anticancer and antioxidant activities. Its ability to scavenge free radicals suggests a protective role against oxidative stress-associated cancer progression while also exerting direct anticancer effects *via* cell cycle arrest and apoptosis induction.

### 2.6. Assessment of tubulin-β activity

**Fig. 8:**
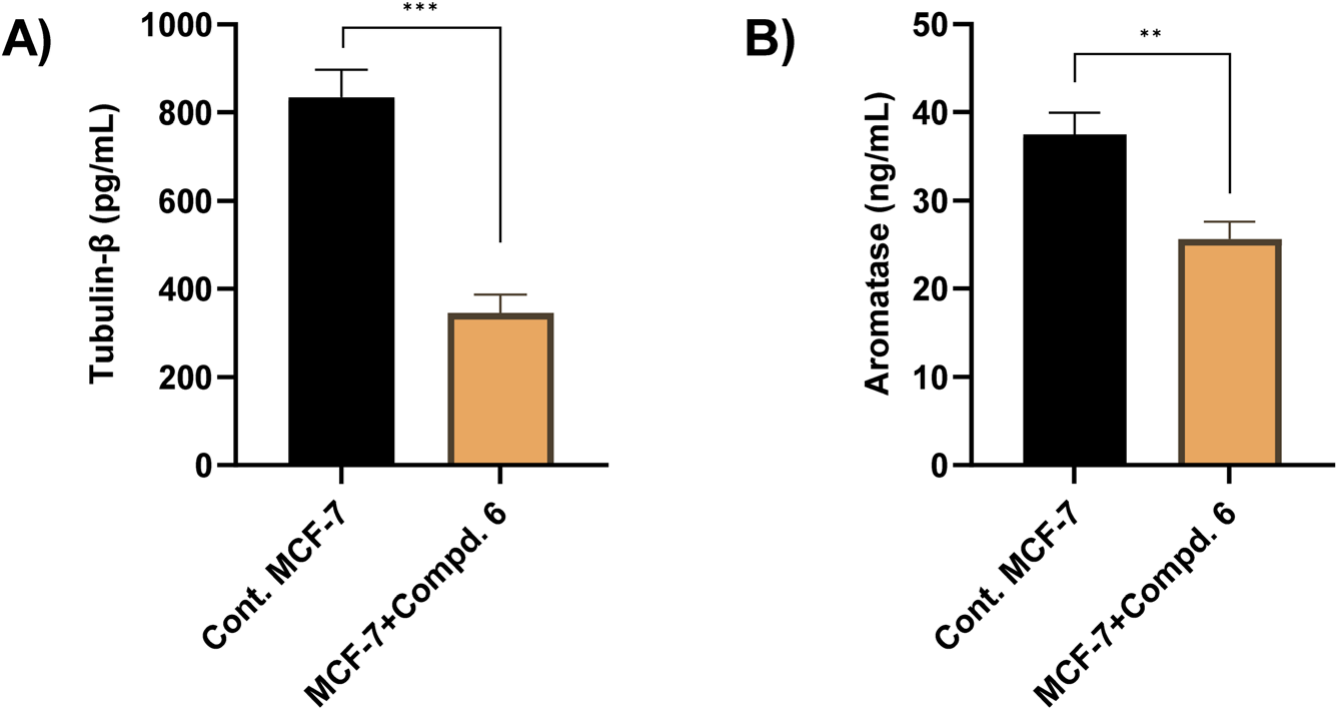
The effect of compound 6 (at its IC_50_ value, 4.23 μM) on the tubulin-β (A) and aromatase (B) enzyme activity. The presented data is displayed in triplicate as mean ± SD.

To investigate whether the antiproliferative activity of compound 6 is associated with its effect on microtubule dynamics, a tubulin-β inhibition enzyme assay was conducted for MCF-7 cells. As shown in Fig. 8, our results demonstrated that the treatment of MCF-7 cells with compound 6 (at its IC_50_ value, 4.23 μM) significantly reduced the tubulin-β concentration from 834.6 pg mL^−1^ in untreated MCF-7 cells to 345.7 pg mL^−1^ in treated cells, representing a 58.46% inhibition. This reduction indicates that compound 6 effectively inhibits tubulin-β, which may contribute to its cytotoxic effect on cancer cells. Align with our findings, several 1,2,4-triazole-3-thione derivatives showed a potential inhibitory activity toward tubulin polymerization, supporting the notion that triazole-based structures may interact with tubulin-binding sites.^53–55^ Given that tubulin-β is a key structural component of microtubules, which are essential for cell division and mitotic progression, its inhibition can lead to disruption of the mitotic spindle, cell cycle arrest, and subsequent apoptosis.^56,57^ Thus, our findings suggest that the antiproliferative effects of compound 6 are linked to its inhibition of tubulin-β polymerization.

### 2.7. Assessment of aromatase activity

Aromatase enzyme plays a crucial role in regulating the growth of hormone-dependent breast cancer, particularly by mediating estrogen biosynthesis.^58,59^ In this regard, we envisioned further exploring whether the antiproliferative activity of compound 6 is correlated with its ability to target aromatase. Toward this, an aromatase enzyme inhibition assay was performed as previously reported.^60,61^ Our results revealed that compound 6 (at its IC_50_ value, 4.23 μM) significantly reduced aromatase levels, from 37.52 ng mL^−1^ to 25.64 ng mL^−1^, representing a 31% reduction as compared to the control group (Fig. 8). These results indicate that compound 6 may contribute to estrogen deprivation, thereby limiting estrogen-driven tumor proliferation. Align with several FDA-approved aromatase inhibitors and recently reported triazole-based derivatives, compound 6 possesses 1,2,4-triazole moiety that may bind to the heme iron of aromatase, preventing the conversion of androgens into estrogens.^62–64^ The observed inhibitory potential of compound 6 toward aromatase activity aligns with our previous findings on its ability to target S-phase arrest, and apoptosis induction, together with tubulin and α-glucosidase inhibition in MCF-7 cells. Since estrogen signaling plays a crucial role in cell cycle progression, its inhibition can lead to disruption of mitotic signaling pathways, contributing to cell cycle arrest.^65^ Additionally, estrogen deprivation has been reported to trigger apoptotic pathways by downregulating survival signaling *via* PI3K/Akt. Therefore, the reduction in aromatase levels may contribute to apoptosis by limiting estrogen-dependent survival signals.^66^ Furthermore, several studies have suggested that microtubule inhibitors can indirectly reduce estrogen signaling by disrupting cellular transport mechanisms involved in hormone receptor trafficking.^67^ Together, our findings highlight compound 6 as a potential multi-target antiproliferative agent.

### 2.8. Molecular docking study

To further affirm the efficiency of compound 6 to target aromatase, α-glucosidase, and tubulin proteins, we conducted detailed molecular modeling studies aiming at assessing its binding affinity and interactions toward the targeted proteins. In this regard, the crystal structures of the targeted proteins were carefully checked and selected based on the resolution and nature of the native co-crystallized ligand. The docking protocol was adjusted for each target protein to ensure that the native co-crystallized ligand binds and interacts with the reported essential amino acid residues in the target pocket of the protein.

#### 2.8.1 Molecular docking toward tubulin protein

**Fig. 9:**
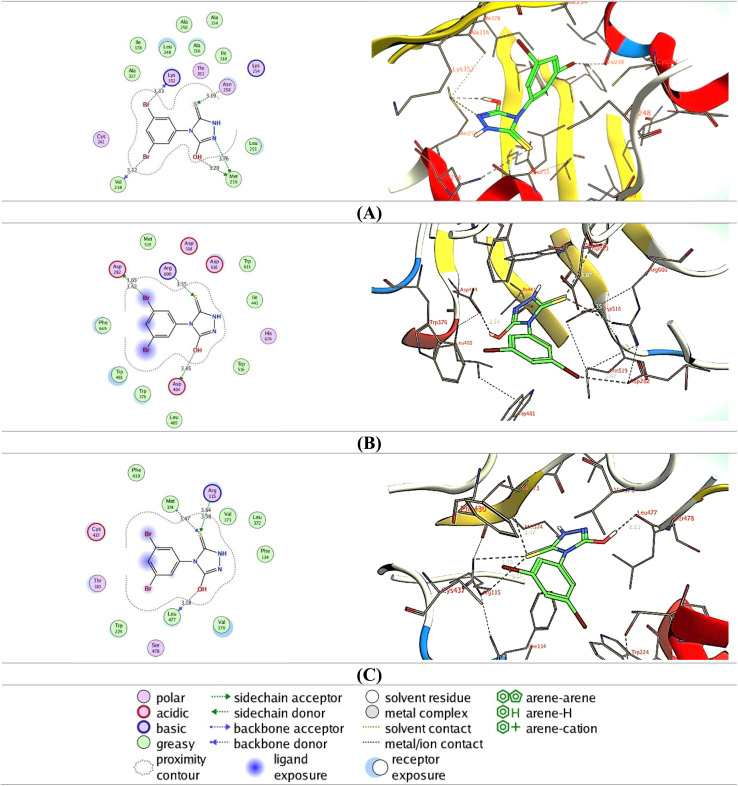
Interaction (2D and 3D) of compound 6 toward the pocket of tubulin (PDB: 4yj2) (A), α-glucosidase (PDB: 5nn8) (B), and aromatase cytochrome P450 (PDB: 5jl6) (C).

In our investigations, we utilized the crystal structure of tubulin in complexation with the potent anticancer agent MI-181 (resolution 2.60 A), which was shown to induce apoptosis and cell arrest.^68^ The validation of the applied protocol, aligned with the reported data, revealed that the MI-181 binds to the pocket of tubulin by forming hydrogen bonds mainly with the two amino acids Val238 and Asn167 residues. Further, the binding of the native ligand was supported by several hydrophobic interactions with grassy amino acid residues (Table 1). The assessment of the molecular modelling of compound 6 indicated that it substantially binds to the active pocket of tubulin by interacting with similar amino acid residues as that shown for MI-181, but also with additional amino acids. As shown in Fig. 9, compound 6 showed the ability to interact *via* its 5-hydroxy-triazole-3-thione moiety with Met259 and Asn258 residues forming 3 hydrogen bonds. Further, the 3,5-dibromophenyl moiety showed hydrophilic interaction with the Val238 and Lys352 residues *via* the two bromo-substituents. Similar to MI-181, the binding affinity of compound 6 was enhanced by the hydrophobic interactions with amino acids including Leu248, Ala250, Leu255, Ala316, Ala317, Ile318, Ala354, and Ile378 residues. These results underscore the capability of compound 6 to target the tubulin activity, supporting our biochemical findings.

**Table 1:** The binding score and interactions of compound 6 and native co-crystallized ligand toward the tubulin, α-glucosidase, and aromatase cytochrome P450

Receptor	Compound	Score (kcal mol^−1^)	Type of interactions	
			Hydrophilic	Hydrophobic
Tubulin (PDB: 4yj2)	MI-181	−9.61	Val238, Asn167	Phe169, Leu248, Leu252, Leu255, Met259, Ala316, Ala317, Ile318, Ala354, Ile378
	Compound 6	−10.05	Asn258, Met259, Val238, Lys352	Leu248, Ala250, Leu255, Ala316, Ala317, Ile318, Ala354, Ile378
α-Glucosidase (PDB: 5nn8)	Acarbose	−13.47	Asp616, Asp282, Arg600, Met519, Asp518, Asp404, His674	Trp376, Leu405, Ile441, Trp481, Trp516, Trp613, Trp618, Phe649, Leu650
	Compound 6	−11.22	Asp282, Arg600, Asp404	Trp376, Leu404, Ile441, Trp481, Trp516, Met519, Phe549, Trp613
Aromatase (PDB: 5jl6)	4-Androstene-3,17-dione	−12.17	Arg115, Met374, Ala306	Ile133, Phe134, Phe221, Trp224, Ile305, Val370, Leu372, Val373, Leu477
	Compound 6	−11.56	Arg115, Met374, Leu477	Phe134, Trp224, Val370, Leu372, Val373, Phe430

#### 2.8.2 Molecular docking toward α-glucosidase protein

We further performed a molecular docking study for compound 6 to assess its binding affinity toward the binding pocket of the α-glucosidase protein. Toward this, we have utilized the crystal structure of α-glucosidase in complex with acarbose, a well-known antidiabetic drug that exhibits potential antitumor activity toward breast cancer (resolution 2.45 A).^69^ Our analysis revealed that acarbose binds with the pocket of α-glucosidase by interacting with 7 essential amino acid residues (Asp616, Asp282, Arg600, Met519, Asp518, Asp404, His674), along with a network of hydrophilic interactions with 9 amino acid residues (Table 1). These results were in agreement with the reported data.^69^ Accordingly, we conducted molecular modelling for compound 6 utilizing the adjusted protocol. Our findings indicated that compound 6 displays a considerable binding affinity toward the active pocket of α-glucosidase. Compound 6 showed the ability to interact with 3 essential amino acid residues (Asp282, Arg600, Asp404) in the active site of the protein. As indicated in Fig. 9, the 5-hydroxy and 3-thione groups of the 5-hydroxy-triazole-3-thione moiety could form two hydrogen bonds with Arg600 and Asp404 residues, respectively. Furthermore, one of the bromo-substituents at the 3,5-dibromophenyl moiety interacted with the Asp282 residue. The affinity of compound 6 was supported by a set of hydrophobic interactions with amino acid residues including Trp376, Leu404, Ile441, Trp481, Trp516, Met519, Phe549, and Trp613. These results suggest the ability of compound 6 to bind to α-glucosidase in similar manner as the native ligand which further supports the detected substantial *in vitro* inhibitory activity.

#### 2.8.3 Molecular docking toward human aromatase protein

Finally, we explored the binding affinity of compound 6 toward the active site of the human aromatase enzyme. In this regard, we conducted our assessment utilizing the crystal structure of aromatase in complex with 4-androstene-3,17-dione, a non-steroidal inhibitor (resolution 3.00 A).^70^ As indicated in Table 1, the binding of the native 4-androstene-3,17-dione ligand is mainly based on three hydrogen bonding interactions with the amino acids Arg115, Met374, and Ala306. The binding of the native ligand was further supported by the hydrophilic interactions with the amino acids Ile133, Phe134, Phe221, Trp224, Ile305, Val370, Leu372, Val373, and Leu477 in the pocket.^70^ The assessment of the binding affinity of compound 6 revealed that it displays a substantial binding score toward the aromatase pocket. The analysis of the binding poses indicated that the binding of compound 6 is mainly based on the 5-hydroxy-triazole-3-thione moiety. As indicated in Fig. 9, compound 6 exhibited the ability to interact *via* its 3-thione group with the essential amino acid residues, including Arg115 and Met374 residues, forming three hydrogen bonds. Further, the 5-hydroxyl group showed H-bonding interaction with Leu477 residue. A network of hydrophilic interactions has been also detected with Phe134, Trp224, Val370, Leu372, Val373, and Phe430 residues that provided the high affinity of compound 6. These findings indicate that compound 6 exhibits a considerable ability to target aromatase enzyme, aligning with its *in vitro* inhibitory activity. Taken together, our extensive molecular docking studies underscore the binding affinity of compound 6 toward the examined targets and support the observed *in vitro* potential of this compound.

### 2.9. Molecular dynamic study

**Fig. 10:**
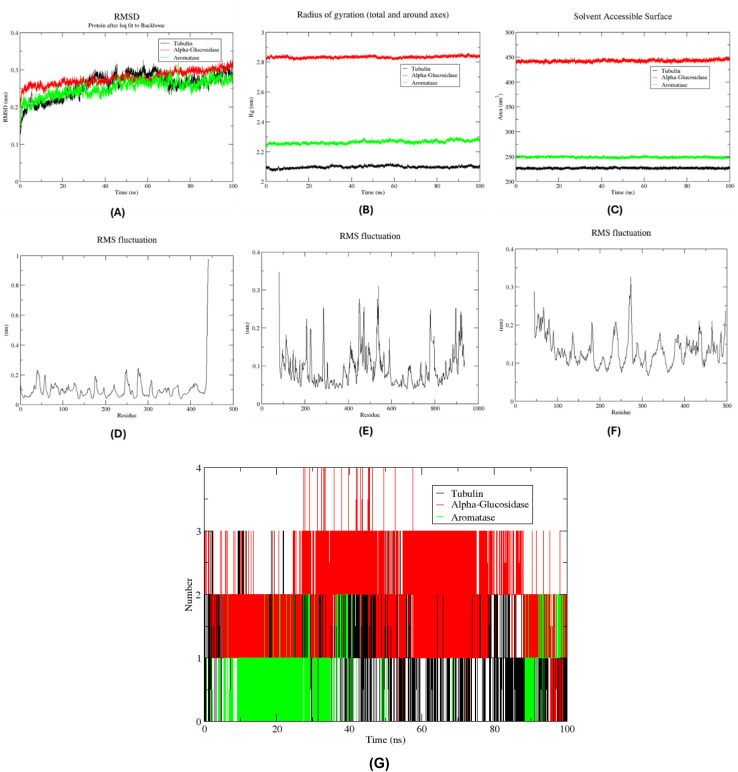
Structural molecular dynamic analysis of compound 6 against the tubulin, α-glucosidase, and aromatase cytochrome P450 showing RMSD (A), radius of gyration (B), SASA values (C), and the Root Mean Square fluctuation (RMSF) of protein backbone; tubulin (D), α-glucosidase (E), aromatase cytochrome P450 (F), and number of H-bonds between compound 6 and the 3 target proteins (G) calculated during the 100 ns of MD trajectories.

To evaluate the conformational stability and dynamic behavior of the protein-ligand complexes, a 100 ns molecular dynamics (MD) simulation was conducted for compound 6 in complex with three different target proteins: tubulin, α-glucosidase, and aromatase cytochrome P450 (Fig. 10). Post-processing of the simulation trajectories was performed using the trjconv tool in GROMACS, involving re-centering and re-wrapping of the complex within the simulation box to eliminate artifacts due to periodic boundary conditions. Subsequent analyses included the calculation of root mean square deviation (RMSD), radius of gyration (*R*_g_), and solvent-accessible surface area (SASA) to assess the stability, compactness, and solvent exposure of the protein structures over the simulation period. The RMSD profiles of the protein backbone atoms revealed that all three complexes attained equilibrium relatively early in the simulation and maintained structural stability throughout the 100 ns trajectory (Fig. 10A). The α-glucosidase–compound 6 complex demonstrated the highest degree of stability, exhibiting minimal fluctuations with an average RMSD below 0.1 nm, indicative of a highly stable binding mode. Similarly, the aromatase and tubulin complexes maintained consistent RMSD values with minor deviations, all within the accepted range for a stable MD simulation. These findings suggest that compound 6 adopts a stable binding pose in the active sites of all three target proteins, with the α-glucosidase complex displaying the most rigid conformation. To further support the structural integrity of the protein complexes, the radius of gyration (*R*_g_) was analyzed to monitor changes in the compactness of the protein backbone (Fig. 10B). A relatively constant *R*_g_ value across the trajectory for each complex indicates that no major unfolding or conformational drift occurred during the simulation. The data showed that all three protein-ligand complexes retained high levels of compactness, supporting the inference that compound 6 binding does not destabilize the global structure of the proteins. In addition, solvent-accessible surface area (SASA) analysis was carried out to monitor variations in the exposure of the protein surfaces to the solvent environment (Fig. 10C). Stable SASA values throughout the simulation period for all three complexes further validated the conformational stability of the protein structures. The absence of significant fluctuations in SASA suggests that the protein–ligand interactions did not induce substantial structural rearrangements that would otherwise alter solvent exposure. Together, the RMSD, *R*_g_, and SASA analyses converge to indicate that compound 6 binds to tubulin, α-glucosidase, and aromatase in a stable and favorable manner over the course of the simulation. The consistently low RMSD values, high structural compactness, and stable solvent accessibility suggest that compound 6 forms persistent interactions with the respective binding pockets, particularly with α-glucosidase.

To gain deeper insights into the flexibility and local dynamics of the protein residues during the simulation, root mean square fluctuation (RMSF) values were calculated for the backbone atoms of each complex over the 100 ns trajectory. RMSF analysis provides residue-level resolution of atomic motion and is particularly informative for identifying flexible loop regions and rigid binding interfaces. As depicted in Fig. 10D–F, all three protein-ligand complexes exhibited relatively low fluctuations across the majority of their residues, indicating overall structural rigidity. Notably, residues directly involved in ligand binding showed minimal fluctuation (<0.2 nm), suggesting a high degree of local stability at the interaction interface. For the tubulin–compound 6 complex (Fig. 10D), critical binding site residues including Asn258, Met259, Val238, and Lys352 exhibited very low RMSF values. Similarly, in the α-glucosidase complex (Fig. 10E), residues Asp282, Arg600, and Asp404 demonstrated restricted mobility, while the aromatase cytochrome P450 complex (Fig. 10F) revealed that Arg115, Met374, and Leu477 remained highly stable throughout the simulation. Importantly, these residues correspond to key interaction points previously identified during molecular docking studies, reinforcing the consistency and reliability of the simulation results. In addition to structural fluctuation analysis, hydrogen bonding patterns were investigated to assess the stability and nature of the intermolecular interactions formed between compound 6 and the protein targets. The number of hydrogen bonds formed throughout the 100 ns trajectory was calculated and plotted over time (Fig. 10G). Compound 6 consistently formed stable hydrogen bonds with all three targets, with particularly strong and persistent interactions observed in the α-glucosidase complex. On average, this complex maintained 2–3 hydrogen bonds throughout the simulation, indicating a well-defined and stable binding mode. In contrast, the tubulin and aromatase complexes showed slightly fewer hydrogen bonds, typically maintaining 1–2 bonds; however, these interactions were still persistent and contributed to the overall binding stability. These observations suggest that the interaction of compound 6 with the key active site residues of each protein target is not only structurally stable but also functionally relevant. The low residue fluctuations at the binding interfaces, coupled with the presence of stable hydrogen bonds, particularly in the α-glucosidase complex, further emphasize the strong affinity and specificity of compound 6.

To assess the binding interactions of compound 6 with its respective protein targets, we performed a per-residue energy decomposition analysis following a 100 ns molecular dynamics (MD) simulation. Pocket residues were identified for each protein—tubulin, α-glucosidase, and aromatase cytochrome P450—by selecting all amino acids within 5 Å of compound 6 using the gmx select tool in GROMACS. These residues were then defined as individual energy groups to evaluate their short-range coulombic (Coul-SR) interaction energies with the ligand using the gmx energy utility. For the tubulin–compound 6 complex (Fig. S9A†), residues including Asn258, Met259, Val238, Lys352, Leu248, Ala250, Leu255, Ala316, Ala317, Ile318, Ala354, and Ile378 were identified within 5 Å of the ligand. Notably, Asn258, Met259, and Leu255 exhibited the most favorable electrostatic interactions, with the lowest Coul-SR energy values. These residues are consistent with those identified in the initial docking studies, reinforcing their role in stabilizing the compound within the binding site throughout the simulation. In the α-glucosidase complex (Fig. S9B†), the residues forming the binding pocket included Asp282, Arg600, Asp404, Trp376, Leu404, Ile441, Trp481, Trp516, Met519, Phe549, and Trp613. Among these, Asp282 and Asp404 demonstrated the most stabilizing interactions, with strongly negative Coul-SR energies. The consistency of these residues with key contacts observed in docking further supports their involvement in the ligand's binding affinity and stability. The aromatase cytochrome P450 complex revealed a larger set of pocket residues, many of which exhibited favorable electrostatic interactions (Fig. S9C†). Residues such as Arg115, Trp224, and Leu477 showed the strongest interactions, with Coul-SR energies below −5, −7, and −3 kJ mol^−1^, respectively. Val373 and Phe430 also contributed moderately with energies near −2 kJ mol^−1^. Interestingly, only Met374 and Phe134 showed slightly positive Coul-SR energies (less than +2.5 kJ mol^−1^), indicating weak or potentially destabilizing interactions. The dominant contribution of Arg115 to binding stability suggests a strong electrostatic anchoring point within the binding pocket. Overall, the per-residue energy decomposition analysis across all three protein targets confirms the significance of several key amino acids in mediating the stable binding of compound 6. The alignment of MD-based results with docking predictions enhances confidence in these residues as primary contributors to ligand affinity.

## 3. Materials and methods

### 3.1. General information

Analytical-grade solvents including methanol, acetone, ethyl acetate, and ethanol were purchased from Sigma-Aldrich (Germany) and used without further purification. All reagents and chemicals were purchased from TCI, Sigma-Aldrich, and Thermo Fisher (Germany). All melting points were determined using a Reichert hot-stage apparatus and were uncorrected. ^1^H-NMR (400 MHz) and ^13^C-NMR (100 MHz) spectra were recorded on a Bruker Avance DRX 400 MHz spectrometer using deuterated solvents (CDCl_3_, DMSO-d_6_, or CD_3_OD) as specified. FT-IR spectra were recorded on a Shimadzu 470 spectrometer using KBr pellets. Mass spectra were obtained on a Finnigan MAT SSQ-7000 mass spectrometer (ionization method: electron ionization (EI)). Elemental analyses were performed using a 1106 elemental analyzer (Central Services Laboratory, NRC, Cairo, Egypt), with deviations within ±0.5% of theoretical values. MCF-7 and HepG-2 cell lines were obtained from the Regional Center for Mycology and Biotechnology, Al-Azhar University, Cairo, Egypt. Cell culture reagents, including trypsin and antibiotics, were obtained from Lonza (Belgium). Dulbecco's Modified Eagle Medium (DMEM) was purchased from Life Technologies, and fetal bovine serum (FBS) was obtained from HyClone (heat-inactivated, if applicable). Unless otherwise specified, all other reagents and assay kits were obtained from Sigma-Aldrich (Germany).

### 3.2. Synthesis and analytical characteristics

#### 3.2.1 Synthesis of 2-(2-cyanoacetyl)-*N*-phenylhydrazine-1-carbothioamide (3)

To a solution of cyanoacetic acid hydrazide (2) (4.0 g, and 40.4 mmol) in benzene (100 mL), phenyl isothiocyanate (6.3 g, 46.4 mmol) was added. After refluxing for 8 hours, as confirmed by TLC analysis (1 : 1 EtOAc : PE, visualized with 1.3% ninhydrin), the reaction mixture was gradually cooled in an ice bath to 0 °C, resulting in the formation of a yellow solid. The mixture was warmed to ambient temperature and subsequently filtered. The obtained solid product was washed with acetone and dried under a high vacuum. Purification of the crude product by crystallization from ethanol afforded compound 3 as a pale-yellow solid. Yield 78%, m. p.: 181–182 °C. Elemental analysis for C_10_H_10_N_4_OS (M. wt = 234). Calcd: C, 51.27; H, 4.30; N, 23.92. Found: C, 51.18; H, 4.13; N, 23.79. FT-IR (KBr) *ν*_max_ (cm^−1^): 3227 (NH), 2225 (CN), 1689 (C=O), 1605, 1593 (C=C). ^1^H-NMR (DMSO-d_6_, 400 MHz, ppm) *δ*: 3.79 (s, 2H, COCH_2_CN), 7.20–7.51 (m, 5H, Ar–H), 9.83 (br. s, 1H, NH), 10.11 (s, 1H, NH), 10.36 (s, 1H, NH). ^13^C NMR (DMSO-d_6_, 100 MHz, ppm) *δ*: 181.4 (C=S), 162.9 (C=O), 139.3 (N–C–Ar), 128.6, 126.6, 125.9 (C-aromatic), 116.1 (CN), 24.9 (COCH_2_CN).

#### 3.2.2 Synthesis of 5-alkoxy-4-phenyl-2,4-dihydro-3*H*-1,2,4-triazole-3-thione (4, 5)

##### 3.2.2.1 General procedure

2-(2-Cyanoacetyl)-*N*-phenylhydrazine-1-carbothioamide (3) (1.2 g, 5.12 mmol) was dissolved in methanolic or ethanolic sodium hydroxide (620 mg, 15.4 mmol NaOH in 50 mL alcohol) and the obtained mixture was subjected to reflux, with progress monitored by TLC analysis. Following 12–16 hours of stirring under the same conditions, TLC analysis showed the reaction was complete (1 : 3 EtOAc : PE, visualized with KMnO_4_). Upon cooling to 0 °C, the mixture was quenched with water and brought to pH 7.5 using 2% HCl, resulting in solid formation. Following filtration, the solid was rinsed with water and dried. Recrystallization from toluene afforded compounds 4 and 5.

###### 3.2.2.1.1 5-Methoxy-4-phenyl-2,4-dihydro-3*H*-1,2,4-triazole-3-thione (4)

The entitled compound was synthesized starting from compound 3 (1.2 g, 5.12 mmol) by reaction with methanolic sodium hydroxide solution (620 mg, 15.4 mmol NaOH) for 16 h to furnish compound 4 as colorless crystals. Yield 59%, m. p. 95 °C. Elemental analysis for C_9_H_9_N_3_OS (M. wt = 207). Calcd: C, 52.17; H, 4.35; N, 20.29. Found: C, 52.01; H, 4.13; N, 20.08. FT-IR (KBr) *ν*_max_ (cm^−1^): 3260 (NH), 1633 (C=N), 1610, 1586 (C=C), 1025 (C–O). ^1^H-NMR (DMSO-d_6_, 400 MHz, ppm) *δ*: 4.00 (s, 3H, OCH_3_), 7.16–7.66 (m, 5H, Ar–H), 11.13 (s, 1H, NH). ^13^C-NMR (DMSO-d_6_, 100 MHz, ppm) *δ*: 188.9 (C=S), 139.0 (C=N), 138.3 (N–C–Ar), 129.1, 125.3, 123.2, 122.4 (C-aromatic), 58.2 (OCH_3_). ESI-MS (EI, 70 eV) *m*/*z* (%): 207 (M^+^, unstable), 169 (2.56), 167 (100), 166 (45.28), 165 (7.04), 164 (3.51), 136 (4.22), 135 (6.42), 134 (10.48), 133 (13.85), 132 (2.30), 123 (3.19), 122 (4.45), 120 (2.21), 119 (5.67), 118 (14.18) 110 (25.57), 109 (16.38), 108 (3.53) 107 (7.36), 106 (32.29), 105 (14.76), 104 (3.67), 103 (3.48), 93 (2.34), 92 (8.95), 91 (21.27), 90 (3.55), 77 (13.99), 76 (11.97), 75 (12.63), 74 (11.48), 66 (2.36), 65 (14.84), 64 (8.99), 63 (3.64), 58 (3.85), 57 (2.01), 51 (8.14).

###### 3.2.2.1.2 5-Ethoxy-4-phenyl-2,4-dihydro-3*H*-1,2,4-triazole-3-thione (5)

The entitled compound was synthesized starting from compound 3 (1.2 g, 5.12 mmol) by reaction with ethanolic sodium hydroxide solution (620 mg, 15.4 mmol NaOH) for 12 h to afford compound 5 as colorless crystals. Yield 66%, m. p. 105 °C. Elemental analysis for C_10_H_11_N_3_OS (M. wt = 221); Calcd: C, 54.30; H, 4.98; N, 19.00. Found: C, 54.14; H, 4.73; N, 18.83. FT-IR (KBr) *ν*_max_ (cm^−1^): 3225 (NH), 1636 (C=N), 1613, 1589 (C=C), 1067, 1032 (C–O) cm^−1^. ^1^H-NMR (DMSO-d_6_, 400 MHz, ppm) *δ*: 1.30–1.34 (t, 3H, *J* = 8.80 Hz, CH_3_), 4.52–4.56 (q, 2H, *J* = 8.80 Hz, OCH_2_), 7.14–7.65 (m, 5H, Ar–H), 11.06 (s, 1H, NH). ^13^C-NMR (DMSO-d_6_, 100 MHz, ppm) *δ*: 187.8 (C=S), 148.8 (C=N), 138.9, 137.3, 123.5, 122.1 (C-aromatic), 67.6 (OCH_2_), 14.4 (CH_3_). ESI-MS (EI, 70 eV) *m*/*z* (%): 221 (M^+^, 6.85), 220 (M^+^ − 1, 4.01), 218 (4.45), 217 (7.81), 212 (10.52), 208 (4.39), 207 (3.50), 200 (6.70), 199 (7.69), 198 (7.13), 193 (15.23), 192 (4.71), 191 (8.26), 183 (3.02), 182 (9.90), 181 (69.76), 180 (25.90), 179 (25.60), 178 (6.80), 177 (10.14), 171 (5.80), 170 (4.79), 167 (12.75), 161 (8.46), 160 (5.50), 159 (7.48), 153 (20.04), 152 (7.60), 151 (10.10), 148 (17.08), 147 (20.58), 136 (46.00), 135 (99.16), 134 (21.50), 133 (15.47), 128 (8.94), 127 (5.68), 125 (5.97), 124 (6.12), 120 (37.76), 119 (27.64), 117 (10.31), 111 (7.27), 110 (14.43), 106 (15.74), 104 (13.86), 94 (10.94), 93 (100), 92 (28.38), 91 (16.63), 77 (70.77), 75 (4.18), 66 (13.75), 65 (10.41), 64 (7.69), 51 (9.93).

#### 3.2.3 Synthesis of 4-(3,5-dibromophenyl)-5-hydroxy-2,4-dihydro-3*H*-1,2,4-triazole-3-thione (6)

A solution of compound 4 (800 mg, 3.9 mmol) in acetic acid (40 mL) was treated with an aqueous solution of 48% hydrobromic acid (4.7 mL, 39 mmol), followed by tetrabutylammonium bromide (0.1 g, 10 mol%). The reaction was maintained at 110 °C, and its progress was tracked using TLC analysis (1 : 2 EtOAc : PE, visualized with KMnO_4_). After the starting material was fully consumed (12 h), the reaction was cooled to ambient temperature, and carefully diluted with water (100 mL). The mixture was extracted by ethyl acetate (3 × 100 mL) and the mixed organic layers were dried over sodium sulfate, filtered, and concentrated under reduced pressure. The obtained residue was dissolved again in acetic acid (30 mL) at ambient temperature and the resulting mixture was heated to 60 °C. To this mixture, a solution of bromine (1 mL, 11.7 mol) in acetic acid (5 mL) was added dropwise over 20 min. Following 30 minutes of stirring under identical conditions, a further 3 mL of bromine solution was introduced, and the reaction was stirred for an additional 6 hours until completion, as verified by TLC analysis. Subsequently, the reaction mixture was allowed to cool to room temperature and carefully quenched with ice-cold water. The resulting mixture was allowed to stir for 1 h at this condition, during which a yellow solid was precipitated. The obtained solid was isolated by filtration, washed with H_2_O, and dried under vacuum. Finally, the crude solid was purified by recrystallization using EtOH as a solvent to afford compound 6 as yellow crystals. Yield 63%, m. p. 185 °C. Elemental analysis for C_8_H_5_Br_2_N_3_OS (M. wt = 349). Calcd: C, 27.51, H, 1.43; N, 12.03. Found: C, 27.27; H, 1.26; N, 11.79. FT-IR (KBr) *ν*_max_ (cm^−1^): 3423 (br. OH), 3218 (NH), 1631 (C=N), 1605, 1583 (C=C), 1033 (C–O) cm^−1^. ^1^H-NMR (DMSO-d_6_, 400 MHz, ppm) *δ*: 7.88 (s, 1H, Ar–H), 8.05 (s, 2H, Ar–H), 9.78 (s, 1H, NH), 11.10 (s, 1H, OH). ^13^C-NMR (DMSO-d_6_, 100 MHz, ppm) *δ*: 189.9 (C=S), 156.5 (HO–C=), 134.5, 130.9, 116.2, 112.4 (C-aromatic). ESI-MS (EI, 70 eV) *m*/*z* (%): 349 (M^+^, unstable), 293 (3.96), 292 (17.68), 291 (98.62), 290 (41.23), 289 (100), 288 (44.59), 287 (32.79), 276 (5.64), 275 (3.53), 274 (4.45), 273 (7.84), 272 (4.06), 247 (1.37), 246 (7 06), 232 (2.41), 231 (1.32), 211 (3.43), 210 (16.15), 209 (15.81), 208 (8.24), 202 (1.70), 201 (2.89), 200 (5.92), 199 (7.58), 198 (6.44), 195 (4.60), 194 (1.28), 191 (3.48), 182 (5.69) 181 (9.70), 180 (5.54), 179 (7.65), 178 (4.02), 168 (4.16), 167 (20.23), 166 (5.73), 154 (4.38), 153 (3.57), 152 (4.26), 151(3.10), 140 (4.44), 139 (4.51), 130 (2.96), 128 (2.10), 118 (6.69), 117 (3.35), 102 (2.34), 101 (1.24), 92 (2.92), 91 (19.93), 90 (6.75), 89 (7.83), 78 (2.94), 77 (4.59), 76 (5.00), 75 (3.24), 65 (6.40), 64 (2.81), 63 (8.80), 51 (3.78).

#### 3.2.4 Synthesis of 4-phenyl-5-thioxo-1,2,4-triazolidin-3-one (7)

Compound 3 (1.50 g, 6.4 mmol) was treated with 10% aqueous sodium hydroxide (35 mL) at ambient temperature. The mixture was maintained under reflux for 12 hours, with progress monitored by TLC (1 : 3 EtOAc : PE, visualized with KMnO_4_), then cooled to 0 °C. The mixture was then treated with 2% HCl solution till pH 7.5 to provide a solid crude product. The solid obtained after filtration was rinsed with water and dried. Recrystallization from ethanol gave compound 7 as pale yellow crystals. Yield 83%, m. p. 222 °C. Elemental analysis for C_8_H_7_N_3_OS (M. wt = 193). Calcd: C, 49.74; H, 3.63; N, 21.76. Found: C, 49.49; H, 3.42; N, 21.53. FT-IR (KBr) *ν*_max_ (cm^−1^): 3422 (br. OH), 3218 (NH), 1605, 1588 (C=C), 1121, 1063 (C–O) cm^−1^. ^1^H-NMR (DMSO-d_6_, 400 MHz, ppm) *δ*: 6.92–8.05 (m, 5H, Ar–H), 10.10 (s, 1H, NH), 10.70 (s, 1H, NH). ^13^C-NMR (DMSO-d_6_, 100 MHz, ppm) *δ*: 176.5 (C=S), 161.0 (C=N), 134.0, 130.6, 118.8, 111.9, 107.7, 103.3 (C-aromatic). ESI-MS (EI, 70 eV) *m*/*z* (%): 194 (M^+^ + 1, 15.47), 193 (M^+^, 68.38), 184 (6.32), 183 (11.63), 182 (9.37), 181 (31.44), 180 (20.90), 176 (6.86), 175 (13.74), 173 (12.82), 172 (11.82), 167 (8.68), 166 (7.30), 152 (9.94), 151 (10.60), 150 (13.31), 149 (22.54), 145 (10.20), 144 (12.65), 143 (9.83), 142 (7.54), 141 (67.94), 140 (11.59), 136 (18.22), 135 (65.76), 134 (59.07), 133 (12.87), 124 (6.95), 123 (12.48), 121 (7.45), 120 (15.97), 119 (34.20), 118 (5.78), 117 (6.35), 105 (6.88), 104 (14.90), 102 (9.59), 99 (12.64), 97 (14.59), 94 (13.86), 93 (100), 92 (23.52), 91 (34.48), 90 (23.92), 84 (6.20), 83 (18.08), 82 (9.83), 78 (10.17), 77 (49.64), 76 (10.05), 67 (11.02), 66 (24.33), 65 (16.97), 58 (6.35), 57 (7.35), 55 (8.28), 51 (10.31).^71–73^

#### 3.2.5 Synthesis of 4-(3,5-dibromophenyl)-5-thioxo-1,2,4-triazolidin-3-one (8)

A stirred solution of compound 7 (600 mg, 3.1 mmol) in glacial CH_3_COOH (30 mL) at 60 °C was treated dropwise with a bromine solution (600 μL, 9.3 mol, in 4 mL glacial CH_3_COOH) over 20 min. Stirring was continued for 40 minutes under the same conditions before adding an additional 2 mL of bromine solution. The reaction mixture was stirred for an additional 10 h at the same conditions (as controlled by TLC analysis; 1 : 3 EtOAc : PE, visualized with KMnO_4_), and subsequently cooled to ambient temperature. Subsequently, the mixture was quenched with 40 mL of ice-cold water and stirred for 1 hour, leading to a solid formation. The solid product was isolated by filtration, thoroughly washed with water, and subsequently dried under vacuum. Compound 8 was finally obtained as pale orange crystals after recrystallization of the crude solid using ethanol as the solvent. Yield 47%, m. p. 179 °C. Elemental analysis for C_8_H_5_Br_2_N_3_OS (M. wt = 349). Calcd: C, 27.51; H, 1.43; N, 12.03. Found: C, 27.30; H, 1.29; N, 11.81. FT-IR (KBr) *ν*_max_ (cm^−1^): 3225 (NH), 1689 (C=O), 1605, 15 916 (C=C) cm^−1^. ^1^H-NMR (DMSO-d_6_, 400 MHz, ppm) *δ*: 7.77 (s, 2H, Ar–H), 8.10 (s, 1H, Ar–H), 9.26 (s, 2H, 2NH). ^13^C-NMR (DMSO-d_6_, 100 MHz, ppm) *δ*: 186.2 (C=S), 167.3 (C=O), 136.4, 133.8, 120.1, and 115.6 (C-aromatic).

### 3.3. Assessment of cellular antiproliferative activity

The cytotoxic activity of the synthesized compounds 4–8 was evaluated against MCF-7 and HepG-2 cells using the 3-(4,5-dimethylthiazol-2-yl)-2,5-diphenyltetrazolium bromide (MTT) assay, following a previously reported method.^26,74–78^ Cells were cultured in a 96-well plate at a density of 1 × 10^5^ cells per well in Dulbecco's Modified Eagle Medium (DMEM) supplemented with 10% FBS and 1% penicillin–streptomycin and under standard conditions (37 °C, 5% CO_2_) overnight. After cell adhesion, a serial dilution of the test compounds 4–8 (dissolved in DMSO, final DMSO concentration ≤0.5% v/v) was added in triplicate, and the cells were incubated for an additional 24 hours under the same conditions. Reference wells were treated with Vinblastine as a standard anticancer drug. The culture medium was discarded after incubation, and the cells were washed once with phosphate-buffered saline (PBS). MTT solution (100 μL, 1 mg mL^−1^ in PBS or culture medium) was added to each well, and the plate was incubated for 4 hours at 37 °C in the absence of light. After incubation, the MTT solution was discarded, and 100 μL of DMSO was added to each well to dissolve the formazan product. The optical density was measured at 570 nm using an ELISA plate reader (SunRise, TECAN, Inc., USA). All experiments were performed in triplicate (*n* = 3), and data were expressed as mean ± SD. Cell viability (%) was calculated relative to the control group, and IC_50_ values were determined using a nonlinear regression model in GraphPad Prism.

### 3.4. Evaluation of α-glucosidase activity

The inhibitory activity of compounds 4–8 against α-glucosidase was assessed using a *p*-nitrophenyl-d-glucopyranoside (*p*NPG) substrate assay, following a previously reported method.^79,80^ In a 96-well plate, a mixture of α-glucosidase (20 μL, 0.5 U mL^−1^, Sigma-Aldrich, Germany) and 150 μL of phosphate buffer (10 mM, pH 6.8) was incubated at 37 °C for 10 minutes. A 20 μL aliquot of the test compounds 4–8 were added at various concentrations (50, 100, 150, 250, 300, and 350 μM), and the reaction was allowed to proceed for 30 minutes at 37 °C. Following this incubation, 20 μL of *p*NPG (1 mM, Sigma-Aldrich, Germany) was introduced into each well, and the reaction mixture was incubated for another 30 minutes at 37 °C. The reaction was terminated by the addition of 50 μL of sodium carbonate (0.1 M solution) to stop enzymatic activity, leading to the formation of a yellow-colored product. Using a UV-Vis spectrophotometer (Spectroquant®, UK), absorbance was recorded at a wavelength of 405 nm. Acarbose was employed as a reference standard, and its inhibitory activity was assessed under identical conditions. The α-glucosidase inhibitory activity (%) was calculated using the following equation:Inhibition (%) = (*A*_control_ − *A*_sample_)/*A*_control_ × 100where *A*_control_ is the absorbance of the reaction mixture without the test compound, and *A*_sample_ is the absorbance in the presence of the test compound or acarbose.

All experiments were performed in triplicate (*n* = 3), and data were expressed as mean ± SD. The IC_50_ values were determined using a nonlinear regression model fitted to the dose–response curve in GraphPad Prism.

### 3.5. Assessment of DPPH radical scavenging activity

The antioxidant activity of compound 6 was evaluated by assessing its efficacy in scavenging the free radicals using 1,1-diphenyl-2-picrylhydrazyl (DPPH) assay, as previously reported.^81–83^ Briefly, a fresh ethanolic solution of DPPH (0.5 mM) was prepared prior to each experiment. A series of concentrations of compound 6 (1, 2.5, 5, 10, 20, 30, 40, and 50 μM in ethanol) were added to a freshly prepared ethanolic DPPH solution (0.5 mM), followed by gentle shaking for 10 minutes. The reaction mixture was then incubated at ambient temperature in the absence of light for 30 minutes. After incubation, the absorbance of each mixture was measured at 517 nm using a UV-Vis spectrophotometer (Spectroquant®, UK). Trolox was employed as a reference standard and treated under identical conditions. The experiments were performed in triplicate (*n* = 3), and results were expressed as mean ± SD. The IC_50_ values for DPPH scavenging activity (%) were determined from the dose–response curve using nonlinear regression analysis in GraphPad Prism.

### 3.6. Assessment of cell cycle analysis by flow cytometer

The evaluation of cell cycle distribution was performed using propidium iodide (PI) stain kit (Abcam, USA) following the manufacturer's protocol and previously reported methods.^77,84^ MCF-7 cells (2 × 10^5^ cells per well) were seeded in DMEM and incubated under standard conditions (37 °C, 5% CO_2_) overnight. The cells were then exposed to compound 6 at its IC_50_ concentration (4.23 μM) for 24 hours, with corresponding solvent controls included. Cells were subsequently washed with cold PBS, and harvested by centrifugation. Pellets were fixed in 70% ethanol at −20 °C for 2 hours, followed by PBS washing and centrifugation. The resulting cell pellets were resuspended in a staining solution containing PI and RNase A and incubated at 37 °C for 30 minutes in the dark to allow for RNA degradation and proper DNA staining. Cell cycle distribution was analyzed using a BD FACSCalibur™ flow cytometer (BD Biosciences, USA). Propidium iodide fluorescence was detected on the FL2 channel (585 nm bandpass filter) with 488 nm excitation, and cell cycle phases (G_0_/G_1_, S, and G_2_/M) were analyzed. All experiments were performed in triplicate (*n* = 3), and data were expressed as mean ± SD. Statistical analysis was conducted using GraphPad Prism, with considering *p* < 0.05 statistically significant.

### 3.7. Assessment of programmed cell death by annexin V-FITC/PI assay

Flow cytometry with Annexin V-FITC and PI staining was used to assess programmed cell death, including both apoptosis and necrosis, following a previously reported method with slight modifications.^77,84^ MCF-7 cells (2 × 10^5^ cells per well) were seeded at 37 °C with 5% CO_2_ in DMEM supplemented with 10% FBS, and 1% penicillin–streptomycin and incubated overnight. Cells were exposed to compound 6 at its IC_50_ value of 4.23 μM for 24 hours, with solvent-treated controls processed in parallel. Post-treatment, cells were detached using 0.25% trypsin–EDTA, washed two times with PBS to remove residual media and trypsin, and resuspended in Annexin V binding buffer for further analysis. To stain the cells, 5 μL of Annexin V-FITC and 5 μL of propidium iodide were added to the suspension, followed by a 15-minute incubation at room temperature in the absence of light. Samples were analyzed using a BD FACSCalibur™ flow cytometer (BD Biosciences, USA). Fluorescence signals were detected using the FL1 channel (530 nm bandpass filter) for Annexin V-FITC and the FL2 channel (585 nm bandpass filter) for PI, and quadrant gating was applied to classify early apoptosis (Annexin V+/PI−), late apoptosis (Annexin V+/PI+), necrosis (Annexin V−/PI+), and viable cells (Annexin V−/PI−). All experiments were performed in triplicate (*n* = 3), and data were expressed as mean ± SD. Statistical analysis was conducted using GraphPad Prism, with an unpaired Student's *t*-test, considering *p* < 0.05 statistically significant.

### 3.8. Assessment of tubulin-β activity

The evaluation of β-tubulin activity in MCF-7 cells was performed following a previously reported protocol with slight modifications.^29^ MCF-7 cells were cultured in DMEM supplemented with 10% FBS, under standard conditions (37 °C, 5% CO_2_). Compound 6 was incubated with MCF-7 cells (2 × 10^6^ cells per well) for 24 hours at 37 °C (final concentration of 4.23 μM). Control wells contained the same volume as the vehicle used for compound 6. After incubation, cells were treated with a biotin-conjugated β-tubulin antibody and incubated at 37 °C for 60 minutes. The cells were then treated with avidin–horseradish peroxidase conjugate and allowed to incubate for an additional 60 minutes under the same conditions. The unbound conjugates were removed by washing with PBS before adding TMB-1000 substrate. Following the enzymatic reaction, the optical density was assessed at 460 nm using a spectrophotometer to determine the β-tubulin concentration in cells. All experiments were conducted in triplicate (*n* = 3), and the data were expressed as mean ± SD. Statistical significance was analyzed using GraphPad Prism, with unpaired Student's *t*-test, considering *p* < 0.05 statistically significant.

### 3.9. Assessment of aromatase activity

Aromatase activity was assessed by applying the fluorescein-based assay (Aromatase CYP19A Activity Assay Kit, Biovision, USA, Catalog # K983-100) following the manufacturer's protocol.^60,61^ MCF-7 cells were cultured under standard conditions at 37 °C with 5% CO_2_ and seeded at 2 × 10^6^ cells per well. The cells were washed with ice-cold PBS after incubation, followed by lysis with 500 μL of aromatase assay buffer per well. Lysis was performed by homogenization using a Dounce homogenizer, followed by incubation on ice for 5 minutes. The lysates were centrifuged at 15 000×*g* for 15 minutes at 4 °C, and the supernatant was collected for enzymatic activity assessment. Protein concentration was determined using the Bradford assay to ensure consistent sample loading. The aromatase enzyme in the lysates was suspended in aromatase assay buffer, and the reaction was initiated by adding 2 μL of the NADPH Generating System (100X) per well. Compound 6 was prepared as a solution in acetonitrile and introduced into the reaction mixture (final concentration of 4.23 μM). Following the manufacturer's protocol, the aromatase substrate was prepared and introduced into the reaction. A control reaction was conducted in parallel with the same volume of acetonitrile but without compound 6. The reaction mixtures were incubated for 60 minutes at 37 °C, after which fluorescence emission spectra were recorded using a fluorescence spectrophotometer at Ex/Em = 488/527 nm. All experiments were performed in triplicate (*n* = 3), and results were expressed as mean ± SD. Statistical analysis was conducted using GraphPad Prism, with unpaired Student's *t*-test, with *p* < 0.05 considered statistically significant.

### 3.10. Molecular docking study

The crystal structure of human aromatase cytochrome P450 (CYP19A1) (PDB: 5JKV), tubulin (PDB: 4YJ2), and human α-glucosidase (PDB: 5NN8) were carefully selected, and obtained from the protein data bank (https://www.rosb.org1) based on the nature of the cocrystallized ligand.^68–70^ The selected proteins were prepared for molecular docking, as previously reported,^85–88^ by 3D protonation, removing water molecules, removing extra chains, hydrogen addition, energy minimization, and prediction of the active site utilizing the MOE Software. The 2D structure for compound 6 was acquired by the ChemDraw program, while the 3D structure was obtained from the MOE program. The 3D structure of compound 6 was energy-minimized and geometrically verified prior to its use in this study. Docking was performed using the triangle matcher placement method, and scoring was carried out using both GBVI/WSA dG and London dG functions. The protocol's accuracy was assessed by comparing the predicted binding pose of the original co-crystallized ligand with its experimentally determined conformation. Subsequently, the protocol was applied to evaluate the binding affinity of compound 6 toward the selected targets. The obtained results were analyzed to determine the binding poses and docking score (kcal mol^−1^) using the MOE program.

### 3.11. Molecular dynamics simulation

A 100 ns molecular dynamics (MD) simulation was conducted for compound 6 in complex with three target proteins: tubulin, α-glucosidase, and aromatase cytochrome P450. All simulations were performed using GROMACS version 2023.1. The CHARMM36 force field was employed to generate protein topologies, while the ligand topology for compound 6 was derived using the CHARMM General Force Field (CGenFF) *via* the official CGenFF server. Each protein–ligand complex was solvated in a triclinic dodecahedral simulation box with a minimum distance of 1.0 nm between the protein surface and the box edge to ensure adequate solvation and prevent artifacts from periodic images. The system was solvated using the TIP3P water model, and appropriate numbers of Na^+^ and Cl^−^ ions were added to neutralize the system charge and mimic physiological ionic strength. Energy minimization was carried out using the steepest descent algorithm to relieve steric clashes and optimize initial geometries. The minimization process was performed with a maximum of 50 000 steps and a convergence criterion of 10.0 kJ mol^−1^ nm^−1^. Following minimization, the system underwent two equilibration phases. The first phase (NVT) was carried out under constant number of particles (*N*), volume (*V*), and temperature (*T*) using a modified Berendsen thermostat to maintain the temperature at 300 K. The second phase (NPT) involved pressure coupling using a Parrinello–Rahman barostat to maintain the system pressure at 1 bar. Each equilibration phase was run for 50 000 steps, corresponding to 100 ps, with a 2 fs integration timestep. Subsequently, the production MD simulation was run for 100 ns under periodic boundary conditions, using a leap-frog integrator with a 2 fs time step. Trajectory coordinates were saved every 10 ps for post-simulation analyses. All bond constraints involving hydrogen atoms were applied using the LINCS algorithm, and long-range electrostatic interactions were treated using the Particle Mesh Ewald (PME) method. Temperature and pressure were maintained throughout the production run at 300 K and 1 bar, respectively.

## 4. Conclusion

In this study, a novel series of 4,5-disubstituted-1,2,4-triazol-3-thione derivatives was synthesized and characterized, with their anticancer potential evaluated through integrated *in vitro* and *in silico* approaches. Among the tested compounds, compound 6 demonstrated the most potent antiproliferative activity, exhibiting IC_50_ values of 4.23 μM and 16.46 μM against MCF-7 and HepG2 cells, respectively, comparable to the reference drug vinblastine. Mechanistic investigations revealed that compound 6 induced significant S-phase cell cycle arrest and triggered apoptosis at both early and late stages, along with moderate necrosis, suggesting a strong intrinsic apoptotic response. Biochemical assays confirmed its multi-target activity through inhibition of tubulin-β polymerization (58.5% reduction), aromatase (31% inhibition), and α-glucosidase (IC_50_ = 122.7 μM), indicating disruption of critical cancer-related pathways such as mitosis, estrogen biosynthesis, and glycoprotein metabolism. Furthermore, compound 6 exhibited notable antioxidant activity (DPPH IC_50_ = 25.4 μM), suggesting its ability to mitigate oxidative stress associated with cancer progression. *In silico* molecular docking studies corroborated these findings by revealing strong binding affinities of compound 6 toward the active sites of tubulin, α-glucosidase, and aromatase, supported by hydrogen bonding and hydrophobic interactions with key catalytic residues. Molecular dynamics simulations over 100 ns further validated the structural stability of the compound–protein complexes, particularly with α-glucosidase, showing consistent RMSD, compactness, low residue fluctuation, and stable hydrogen bonding. Energy decomposition analyses identified key residues such as Asn258 (tubulin), Asp282 (α-glucosidase), and Arg115 (aromatase) as principal contributors to binding stability. Collectively, these findings position compound 6 as a promising multitarget anticancer lead with combined cytotoxic, enzyme-inhibitory, and antioxidant properties. Further optimization through SAR studies, alongside *in vivo* efficacy and pharmacokinetic evaluations, is warranted to further explore the therapeutic potential of this scaffold in cancer treatment.

## Author contributions

Conceptualization, H. A. A., G. A., R. A. A., A. T. B., I. A. A. I., and E. M. S.; methodology, H. A. A., G. A., R. A. A., A. T. B., M. A., H. A. A., E. S. Z., I. A. A. I., and E. M. S.; software, H. A. A., G. A., M. A., H. A. A., E. S. Z., I. A. A. I., and E. M. S.; validation, H. A. A., G. A., R. A. A., A. T. B., M. A., H. A. A., I. A. A. I., and E. M. S.; formal analysis, H. A. A., G. A., R. A. A., A. T. B., M. A., H. A. A., E. S. Z., I. A. A. I., and E. M. S.; investigation, H. A. A., G. A., R. A. A., and E. M. S.; resources, H. A. A., G. A., R. A. A., A. T. B., M. A., H. A. A., I. A. A. I., and E. M. S.; data curation, H. A. A., G. A., R. A. A., A. T. B., M. A., H. A. A., E. S. Z., I. A. A. I., and E. M. S.; writing—original draft preparation H. A. A., G. A., R. A. A., A. T. B., M. A., H. A. A., E. S. Z., I. A. A. I., and E. M. S.; writing—review and editing, H. A. A., G. A., R. A. A., A. T. B., M. A., H. A. A., E. S. Z., I. A. A. I., and E. M. S.; visualization, H. A. A., R. A. A., M. A., H. A. A., and E. M. S.; supervision, H. A. A., G. A., R. A. A., I. A. A. I., and E. M. S.; project administration, H. A. A., G. A., R. A. A., A. T. B., I. A. A. I., and E. M. S.; funding acquisition, H. A. A., M. A., I. A. A. I., and E. M. S. All authors have read and agreed to the published version of the manuscript.

## Conflicts of interest

The authors affirm that they are not aware of any personal or financial conflicts that would have seemed to affect the findings of this study's research.

## Supplementary Material

RA-015-D5RA02512E-s001

## Data Availability

Data supporting the results reported in this manuscript are included in this article and as part of the ESI.† The raw data supporting the conclusions of this article will be made available by the authors without any undue reservation.
