# Supplementary material for: Synthesis and multi-target antiproliferative evaluation of novel 1,2,4-triazole-3-thione analogues against breast cancer: in silico and in vitro mechanistic insights
Source: RSC Adv. 2025 Jul 14;15(30):24769–90. doi: 10.1039/d5ra02512e (PMC12257928; doi:10.1039/d5ra02512e)
Supplement: RA-015-D5RA02512E-s001 [file RA-015-D5RA02512E-s001.pdf]

Supplementary Information (SI) for RSC Advances.
This journal is © The Royal Society of Chemistry 2025

Supporting Information

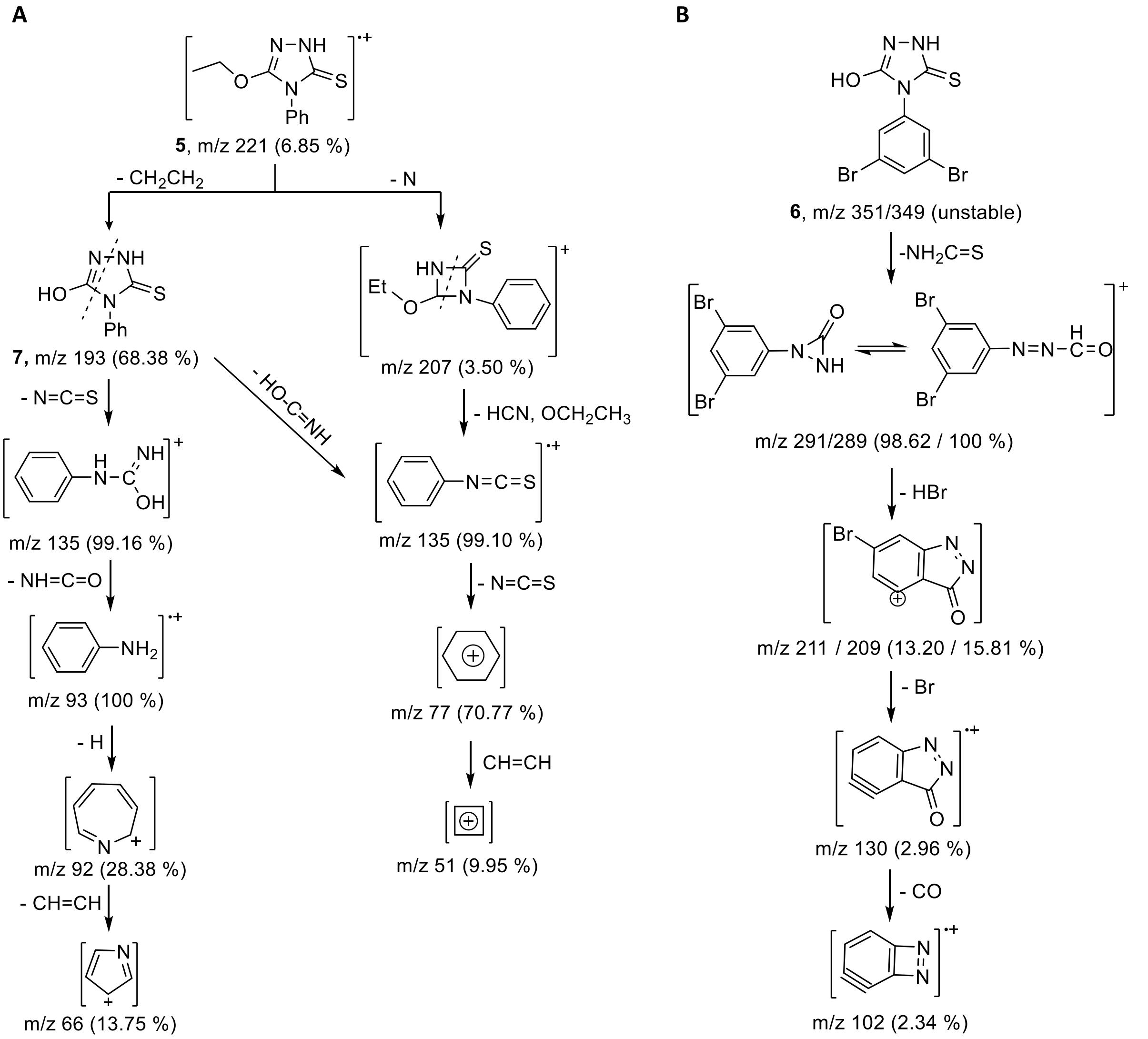

Scheme S1: The mass fragmentation pattern of compounds **5-7**.

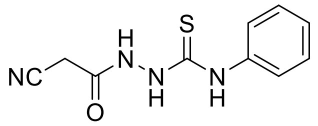

3

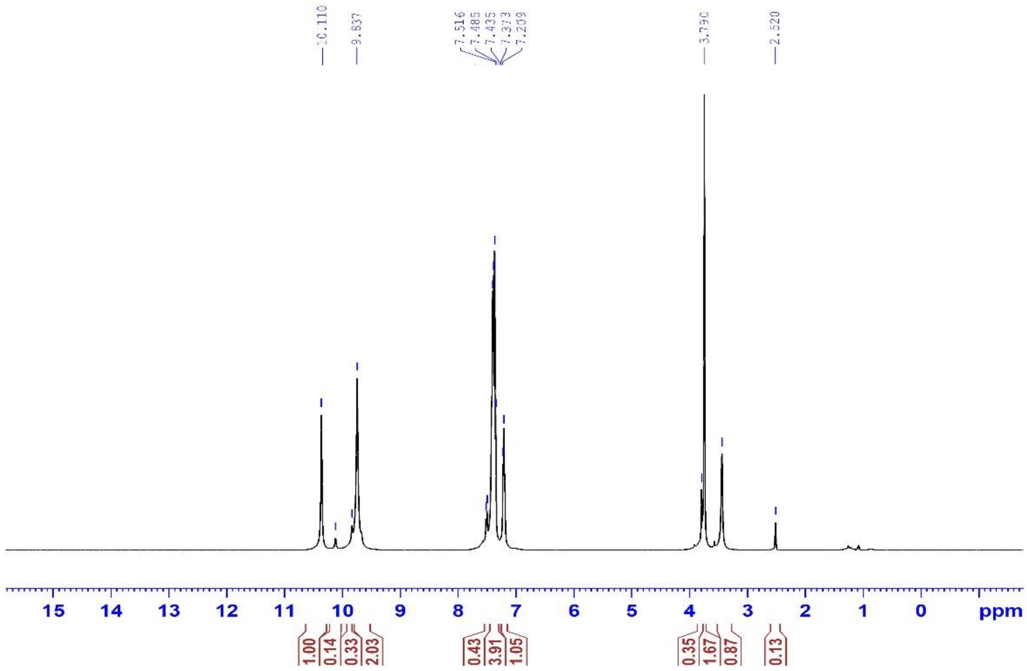

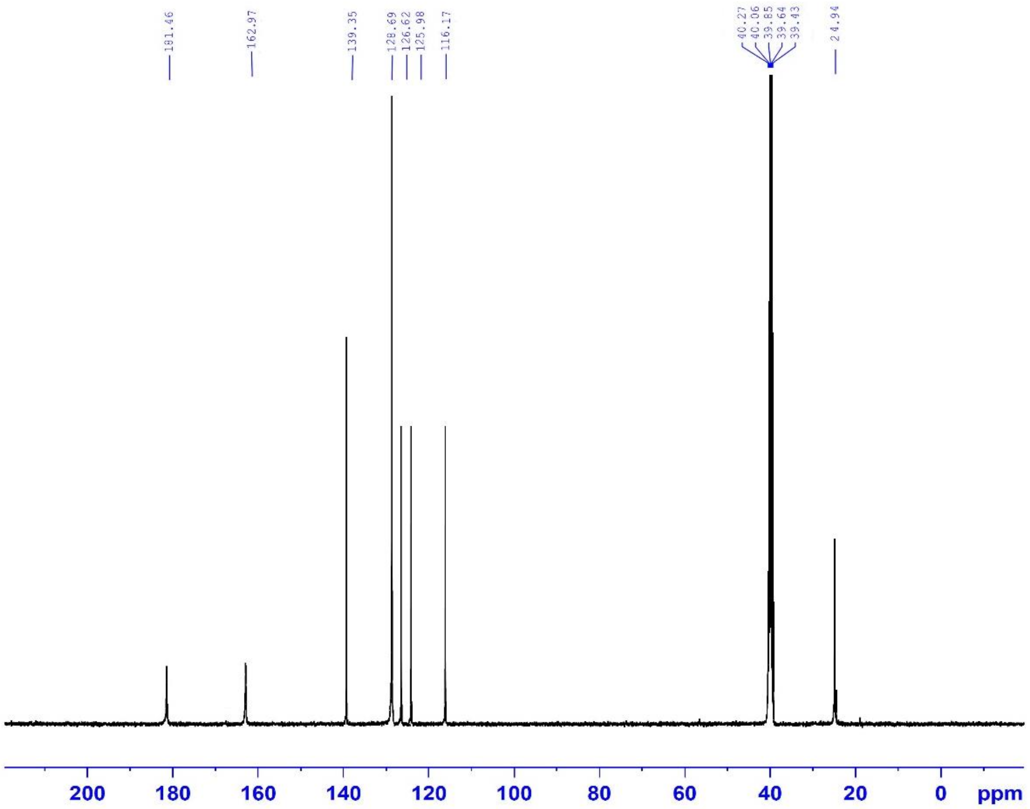

Figure S2: The ^{1}H- and ^{13}C- NMR analysis of compounds **3**.

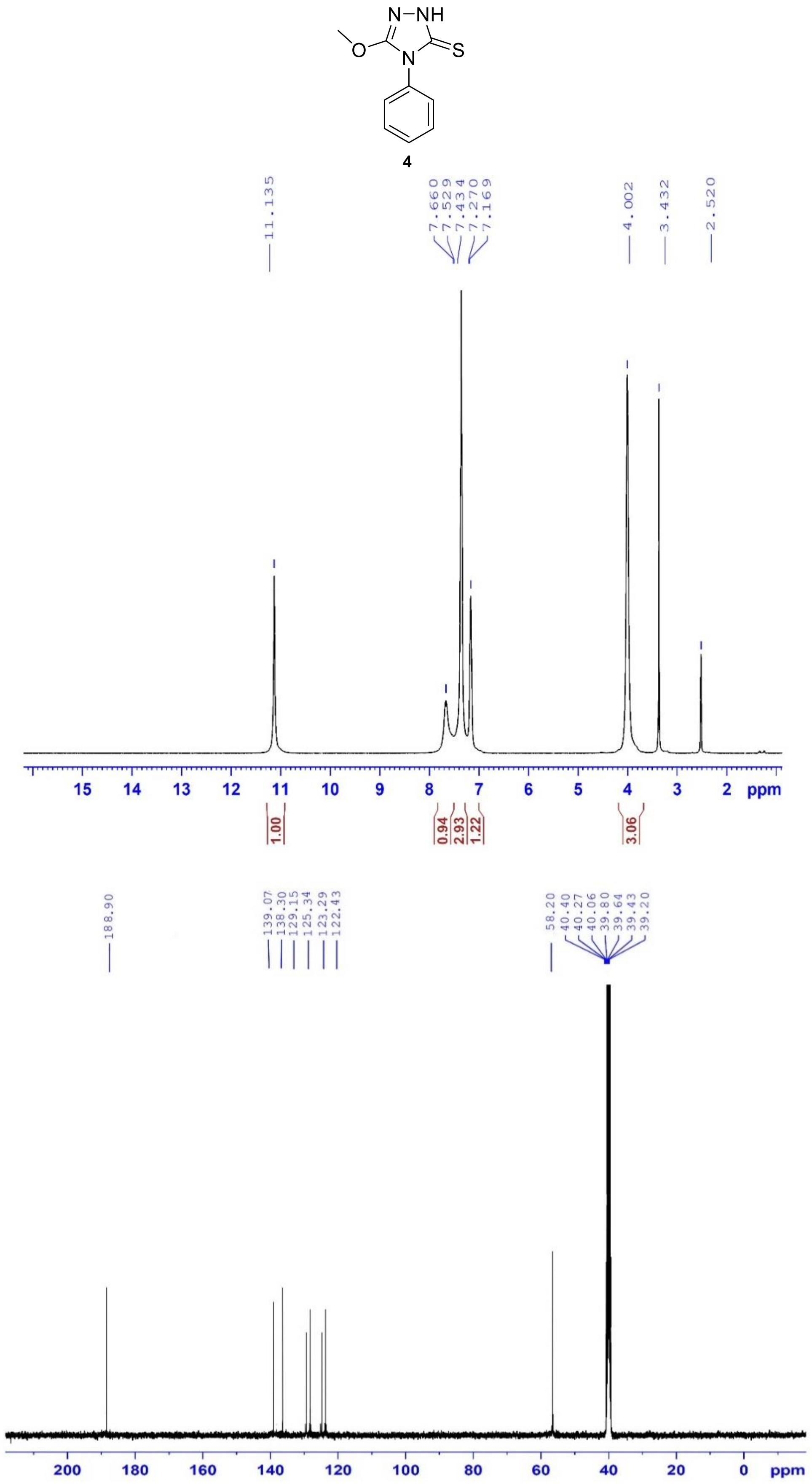

Figure S3: The ^{1}H- and ^{13}C- NMR analysis of compounds **4**.

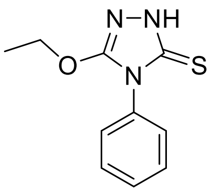
N–NH
O
N
S

5

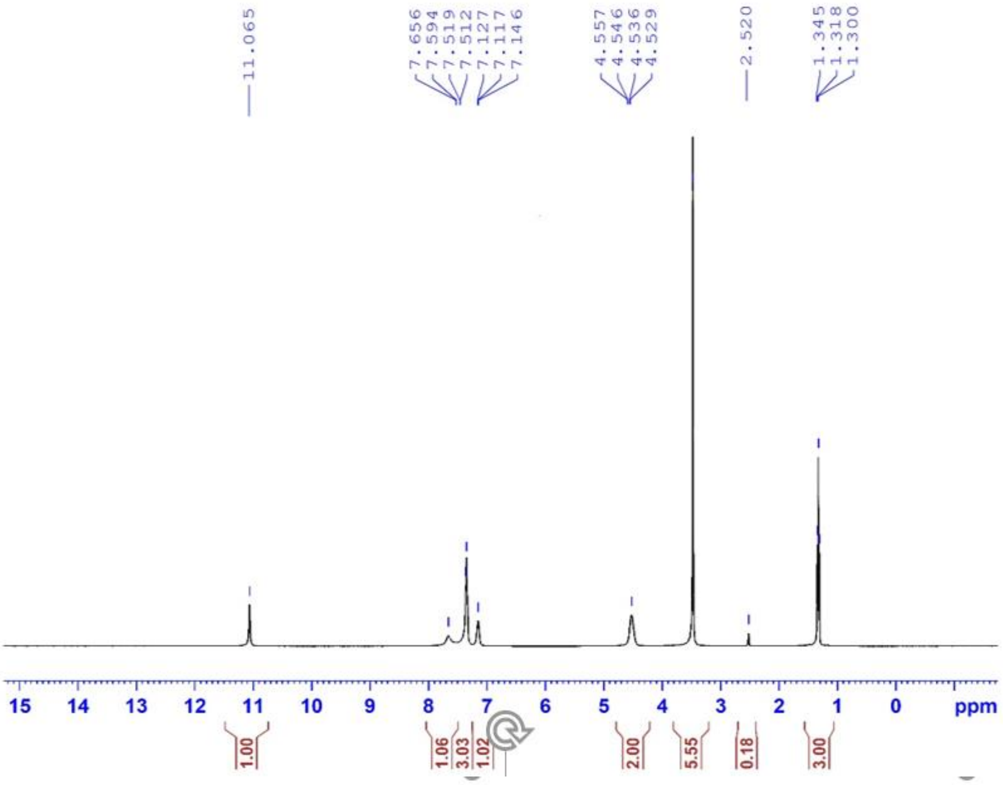
11.065
7.656
7.594
7.519
7.512
7.127
7.117
7.146
4.557
4.546
4.536
4.529
2.520
1.345
1.318
1.300
15 14 13 12 11 10 9 8 7 6 5 4 3 2 1 0 ppm
1.00
1.06
3.03
1.02
2.00
5.55
0.18
3.00

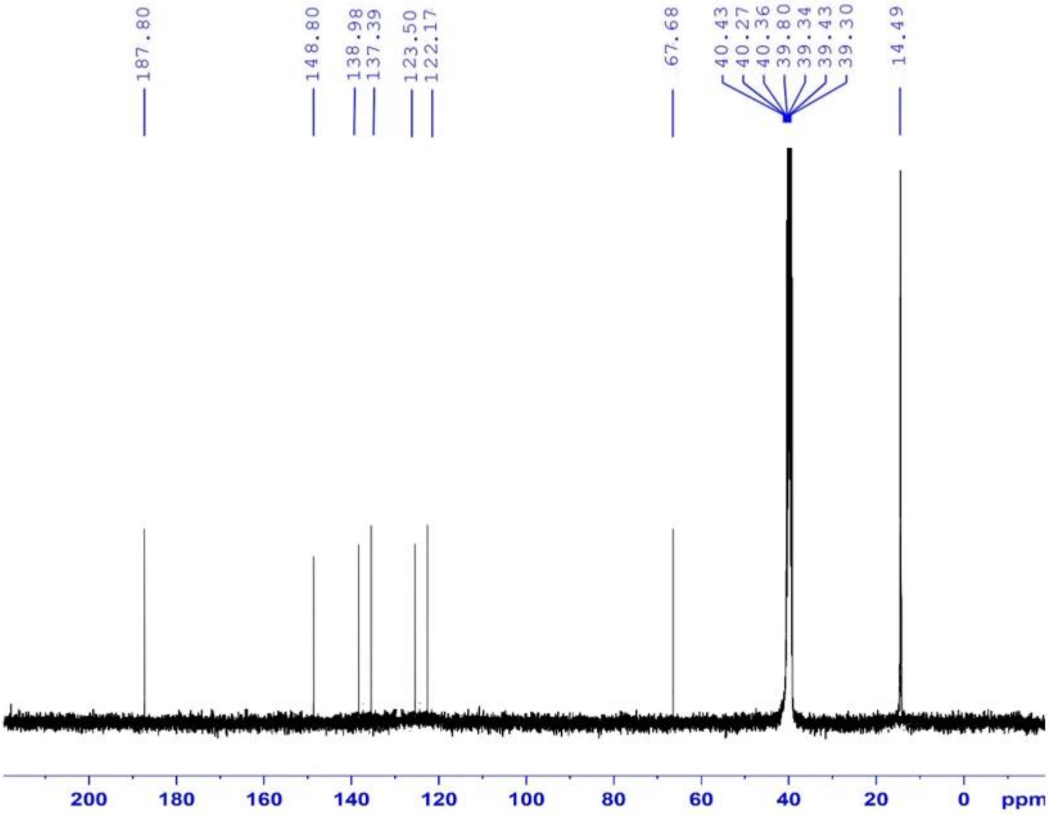
187.80
148.80
138.98
137.39
123.50
122.17
67.68
40.43
40.27
40.36
39.80
39.34
39.43
39.30
14.49
200 180 160 140 120 100 80 60 40 20 0 ppm

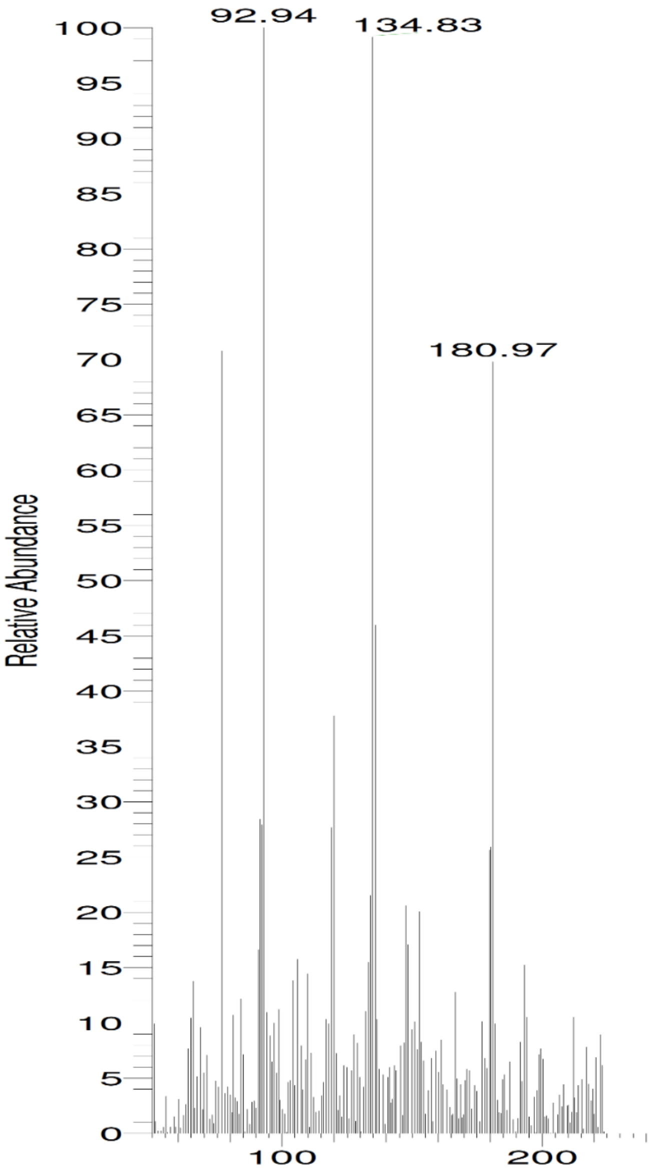

Figure S4: The ^{1}H-, ^{13}C- NMR and mass analysis of compounds **5**.

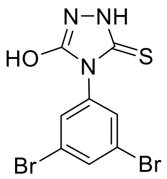
N–NH
HO
N
S
Br
Br

6

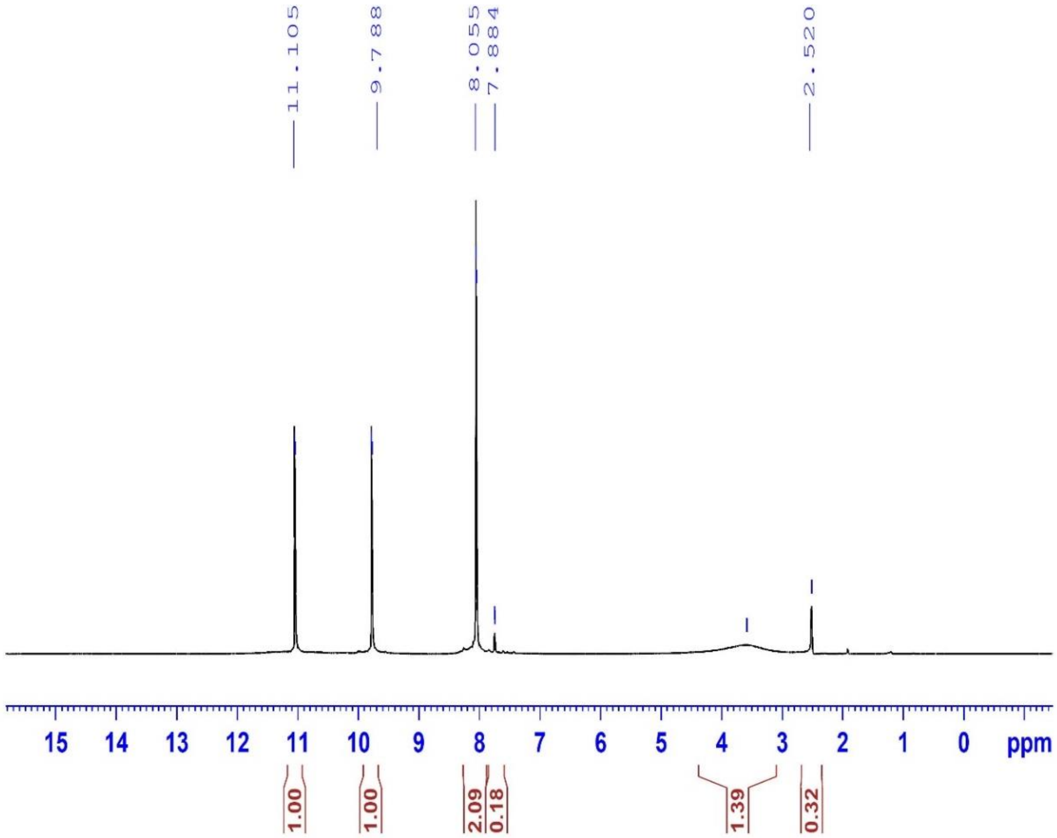
11.105
9.788
8.055
7.884
2.520
15 14 13 12 11 10 9 8 7 6 5 4 3 2 1 0 ppm
1.00
1.00
2.09
0.18
1.39
0.32

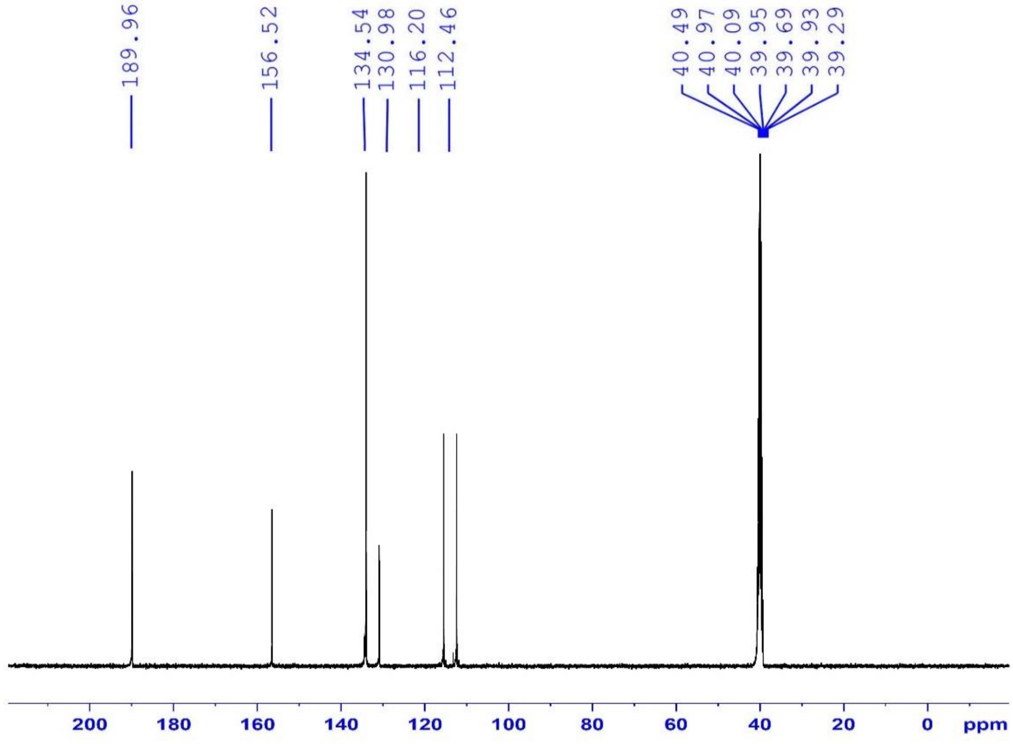
189.96
156.52
134.54
130.98
116.20
112.46
40.49
40.97
40.09
39.95
39.69
39.93
39.29
200 180 160 140 120 100 80 60 40 20 0 ppm

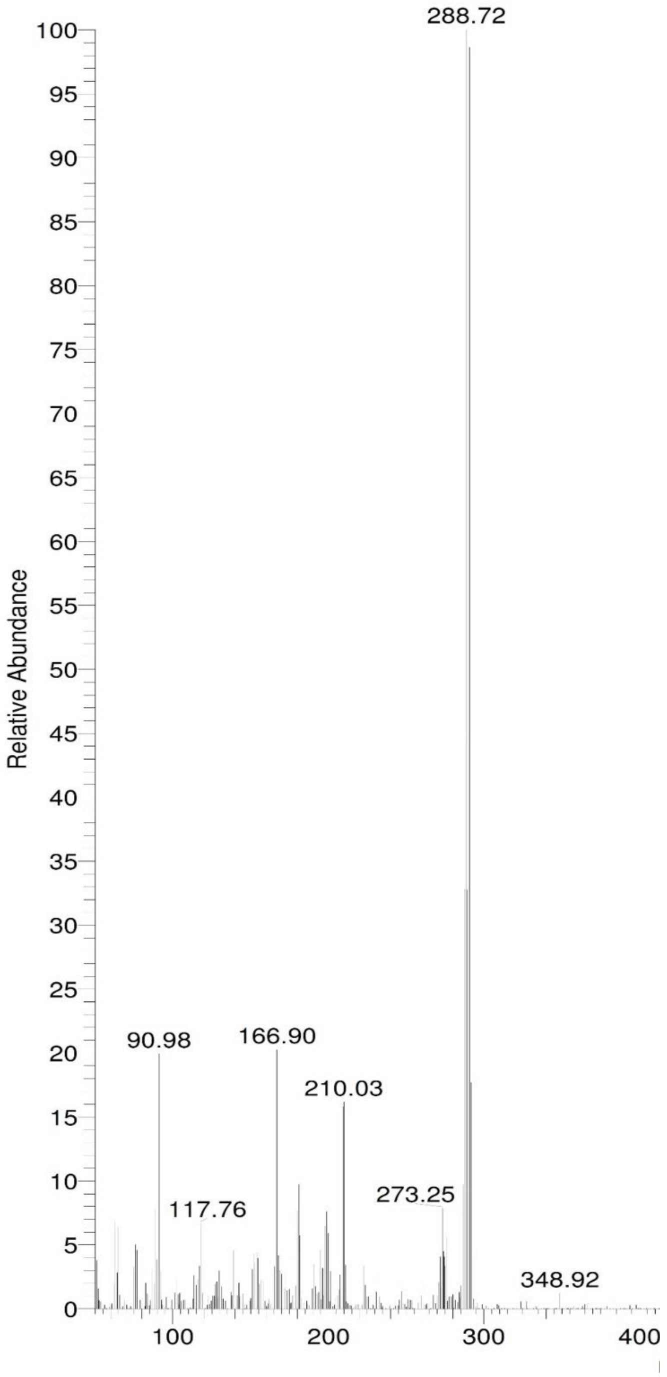

Figure S5: The ^{1}H-,^{13}C- NMR and mass analysis of compounds **6**.

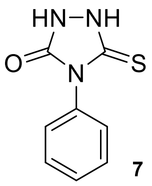

7

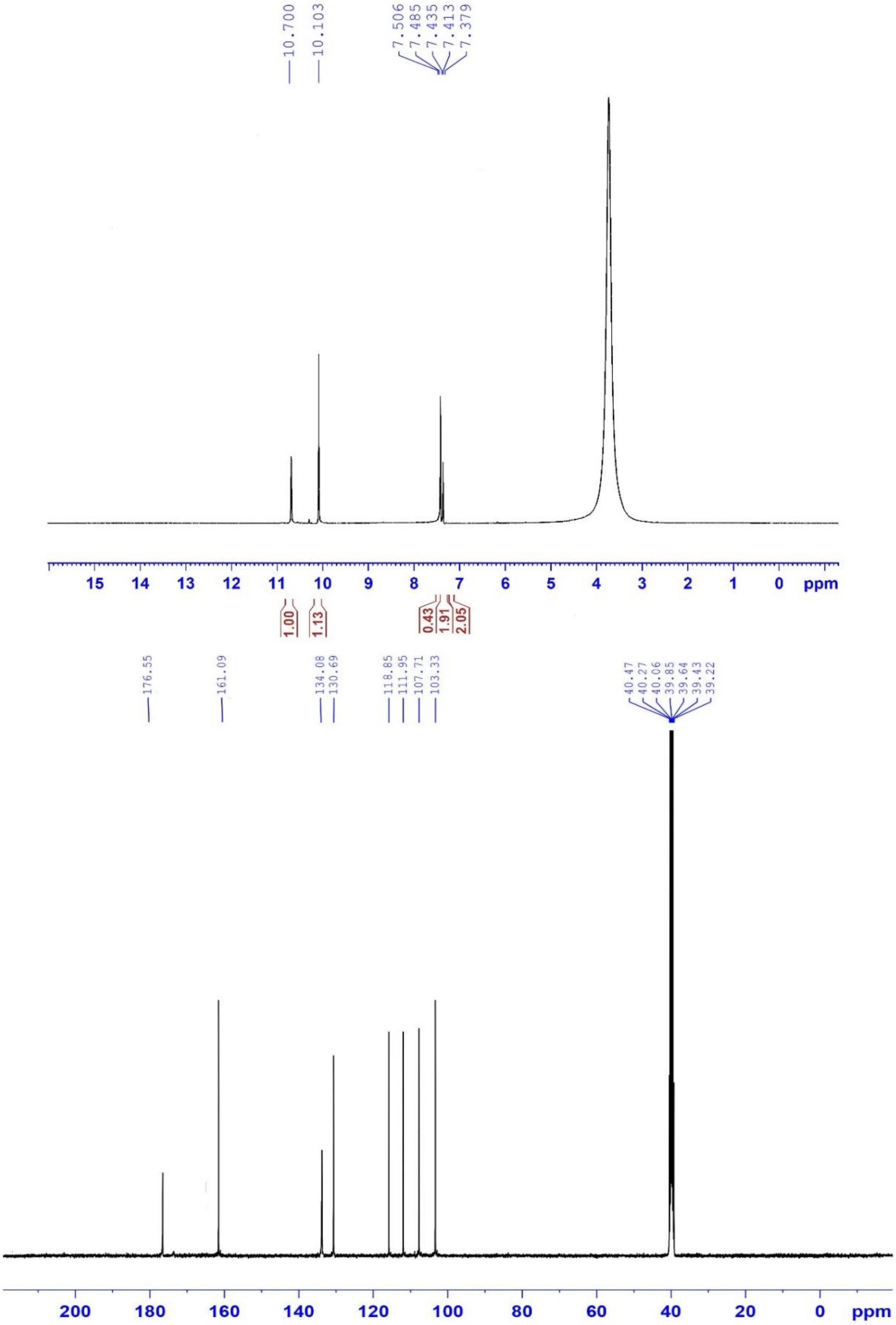

Figure S6: The ^{1}H- and ^{13}C- NMR analysis of compounds **7**.

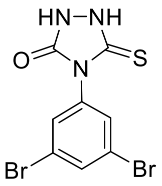
HN–NH
O
N
S
Br
Br

8

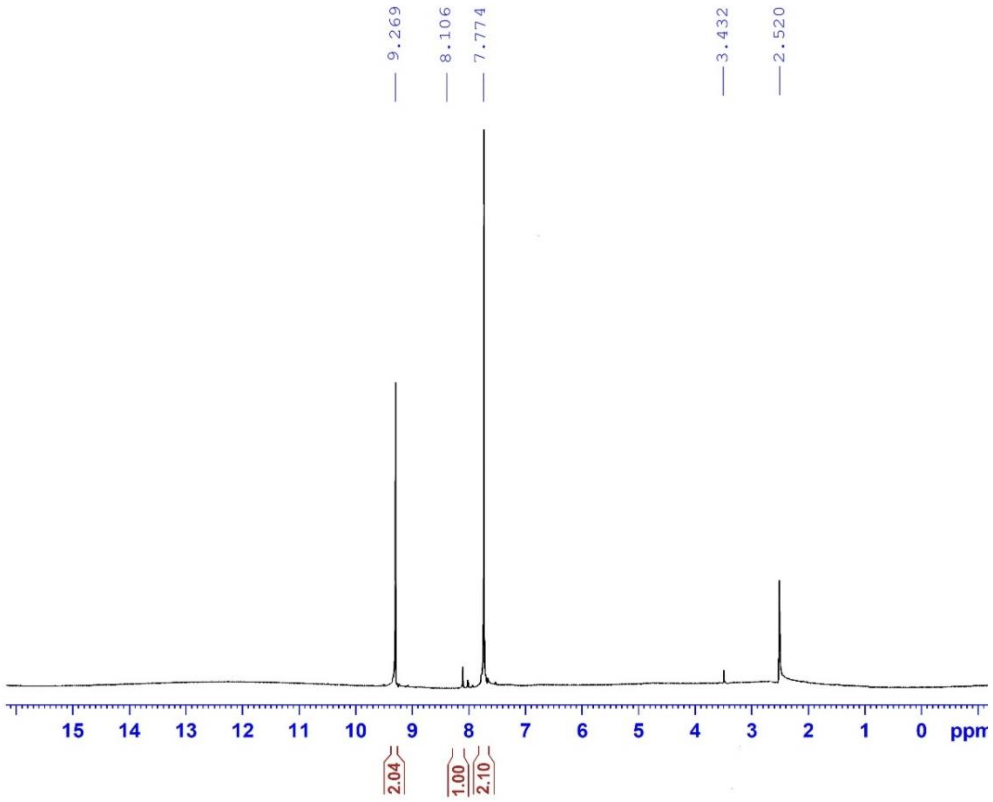
9.269
8.106
7.774
3.432
2.520
15 14 13 12 11 10 9 8 7 6 5 4 3 2 1 0 ppm
2.04
1.00
2.10

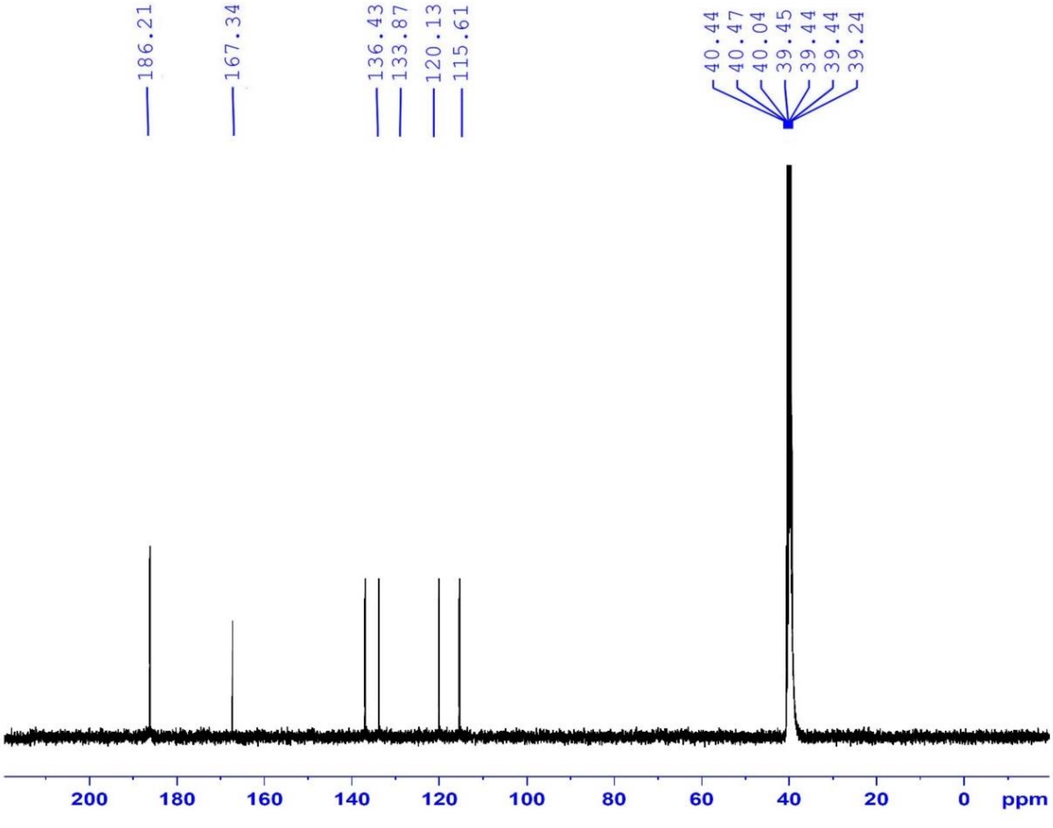
186.21
167.34
136.43
133.87
120.13
115.61
40.44
40.47
40.04
39.45
39.44
39.44
39.24
200 180 160 140 120 100 80 60 40 20 0 ppm

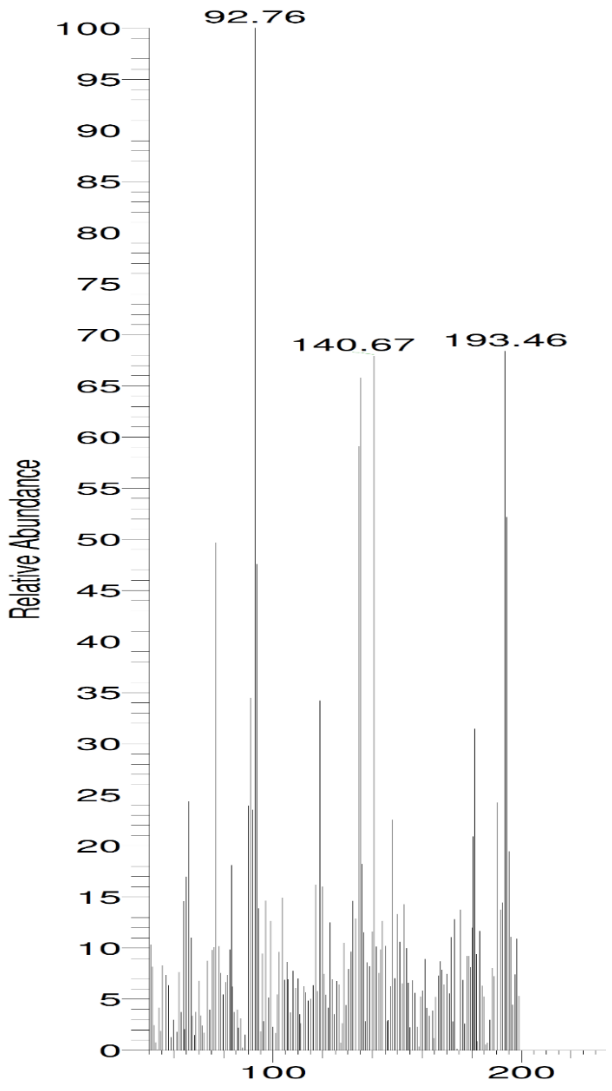

Figure S7: The ^{1}H-,^{13}C- NMR and mass analysis of compounds **8**.

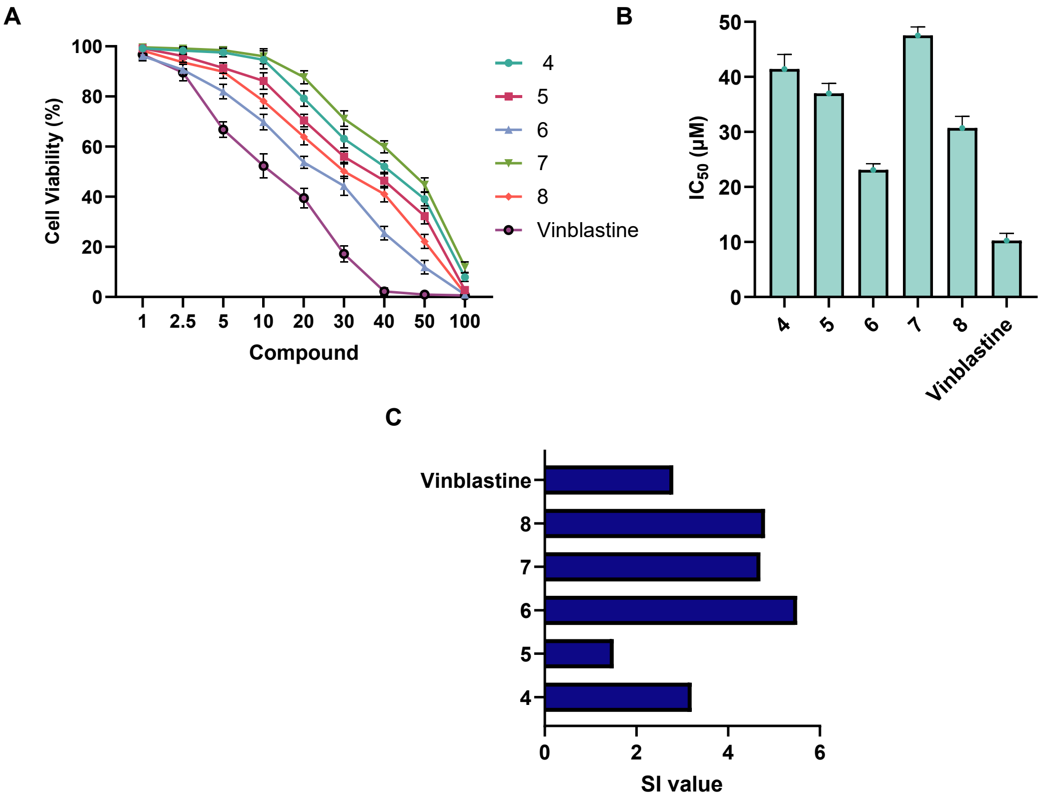

Figure S8. The cytotoxic activity of compounds **4-8** and vinblastine toward the cell viability of MCF-10A cells. **(A)** The dose-dependent activity of compounds **4-8** and vinblastine toward MCF-10A cells. **(B)** The IC_{50} values of of compounds **4-8** and vinblastine toward MCF-10A cells. **(B)** The selectivity index values of compounds **4-8** and vinblastine toward MCF-7 as compared to MCF-10A cells. The presented data is displayed as mean ±SD, n = 3.

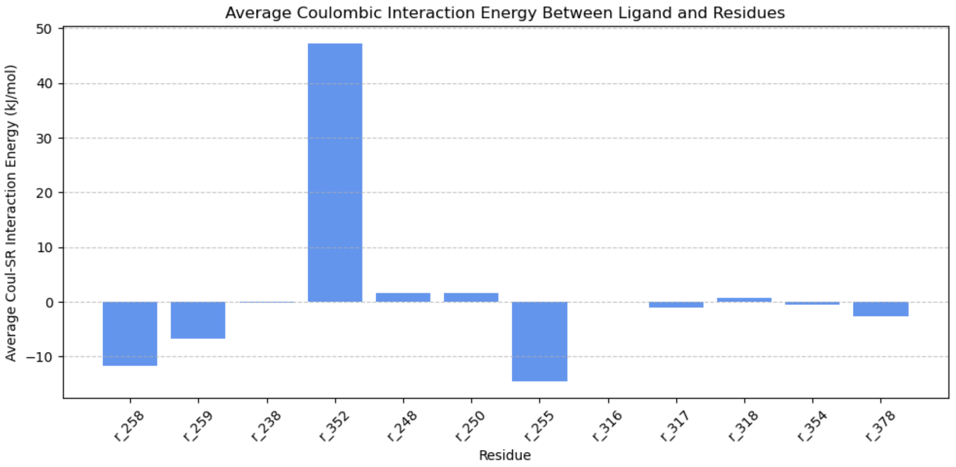

(A)

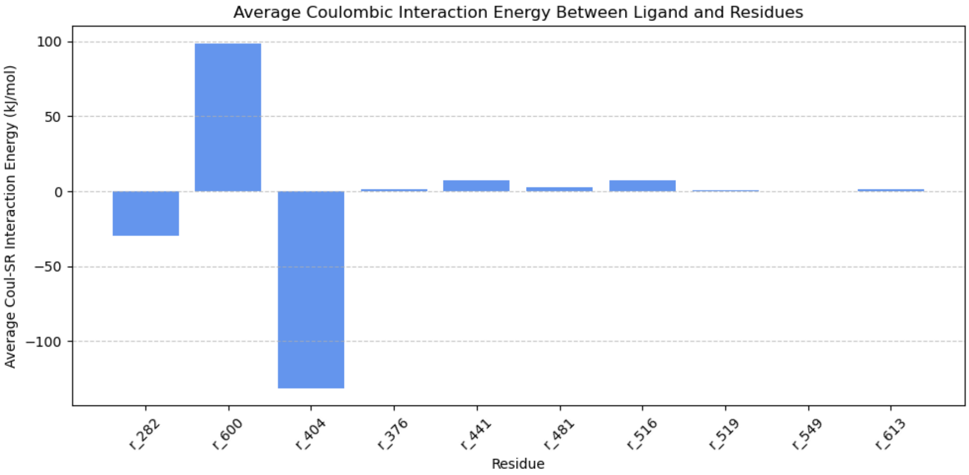

(B)

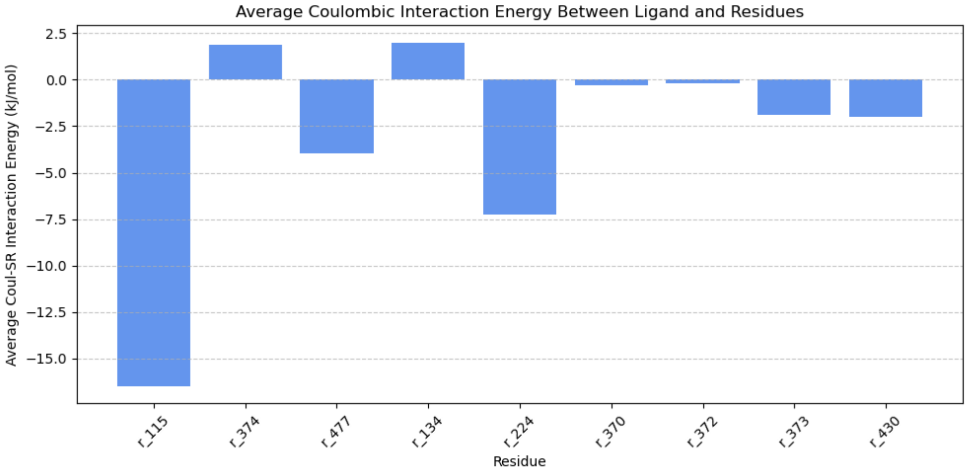

(C)

Figure S9. Average coulombic interaction energy calculated between compound 6 and each pocket residues; tubulin (A), α-glucosidase (B), and aromatase cytochrome P450 (C) calculated during the 100 ns of MD trajectories.
